# Efficacy and safety of 18 anti-osteoporotic drugs in the treatment of patients with osteoporosis caused by glucocorticoid: A network meta-analysis of randomized controlled trials

**DOI:** 10.1371/journal.pone.0243851

**Published:** 2020-12-16

**Authors:** Zhiming Liu, Min Zhang, Zhubin Shen, Junran Ke, Ding Zhang, Fei Yin

**Affiliations:** 1 Department of Spinal Surgery, China-Japan Union Hospital of Jilin University, Changchun, China; 2 Department of Neonatology, Shanghai Children’s Hospital, Shanghai Jiao Tong University, Shanghai, China; Nanjing Medical University, CHINA

## Abstract

**Background:**

Glucocorticoids are widely used in a variety of diseases, especially autoimmune diseases and inflammatory diseases, so the incidence of glucocorticoid-induced osteoporosis is high all over the world.

**Objectives:**

The purpose of this paper is to use the method of network meta-analysis (NMA) to compare the efficacy of anti-osteoporosis drugs directly and indirectly, and to explore the advantages of various anti-osteoporosis drugs based on the current evidence.

**Methods:**

We searched PubMed, Embase and Cochrane Library for randomized controlled trials (RCTs), of glucocorticoid-induced osteoporosis (GIOP) and compared the efficacy and safety of these drugs by NMA. The risk ratio (RR) and its 95% confidence interval (CI) are used as the influence index of discontinuous data, and the standardized mean difference (SMD) and its 95% CI are used as the influence index of continuous data. The statistical heterogeneity was evaluated by the calculated estimated variance (τ^2^), and the efficacy and safety of drugs were ranked by the surface under the cumulative ranking curve (SUCRA). The main outcome of this study was the incidence of vertebral fracture after taking several different types of drugs, and the secondary results were the incidence of non-vertebral fracture and adverse events, mean percentage change of lumbar spine (LS) and total hip (TH)bone mineral density (BMD) from baseline to at least 12 months.

**Results:**

Among the different types of anti-GIOP, teriparatide (SUCRA 95.9%) has the lowest incidence of vertebral fracture; ibandronate (SUCRA 75.2%) has the lowest incidence of non-vertebral fracture; raloxifene (SUCRA 98.5%) has the best effect in increasing LS BMD; denosumab (SUCRA 99.7%) is the best in increasing TH BMD; calcitonin (SUCRA 92.4%) has the lowest incidence of serious adverse events.

**Conclusions:**

Teriparatide and ibandronate are effective drugs to reduce the risk of vertebral and non-vertebral fractures in patients with GIOP. In addition, long-term use of raloxifene and denosumab can increase the BMD of LS and TH.

## Introduction

Glucocorticoids are widely used in a variety of diseases, especially autoimmune diseases and inflammatory diseases, such as rheumatoid arthritis, nephrotic syndrome, systemic lupus erythematosus, inflammatory bowel disease and severe infection and shock. Nearly 1–2% of the world’s people take GCs for a long time, and up to 30–40% of them may have a history of fragile fractures [1], especially the TH, LS and femoral neck fractures [2]. The duration and dose of glucocorticoids can have a serious impact on the risk of fracture. Among the patients who used GCs for a long time, the incidence of fracture (5%) was twice as high as that of those who used GCs for a short time (2.5%) [3]. In addition, the higher the dose, the higher the incidence of fracture. Taking 2.5 mg of prednisone per day will increase the risk of fracture. If the dose is more than 7.5 mg, the risk of fracture will increase as much as 5 times [4].

There are mainly three kinds of anti-osteoporosis drugs: (1) Anti-bone resorption drugs include bisphosphates (such as alendronate, zoledronic acid, risedronate, ibandronate, etidronate and clodronate, etc.), calcitonin (such as elcatonin and salcatonin), selective estrogen receptor modulators (SERMs) (such as raloxifene) and cathepsin K inhibitors. (2) Drugs that promote bone formation include parathyroid hormone analogue (PTHa) (such as teriparatide), active vitamin D and its analogues (such as alfacalcidol and calcitriol); (3) double-acting drugs including strontium salts (such as strontium ranelate) and receptor activator of nuclear factor kappaB ligand (RANKL) inhibitors (such as denosumab). This study will systematically compare the effectiveness and safety of the above-mentioned drugs.

Bisphosphonate is currently the most widely used anti-osteoporosis drug. As an analog of pyrophosphate, it has a strong affinity for hydroxyapatite and can be selectively absorbed and adhered to the mineral surface of bones, resulting in osteoclasts apoptosis, thus exerting an anti-bone resorption effect [5].

Calcitonin drugs mainly reduce bone resorption by inhibiting the number and secretion activity of osteoclasts. Its efficacy is 40–50 times that of human calcitonin, and it can take effect quickly within 2 hours [6].

SERMs play different roles in different tissues. For example, raloxifene can play an estrogen-like effect after binding to the receptor in bone tissue: inhibit bone resorption, increase bone density, and reduce fracture incidence. In the uterus or breast tissue, it presents an estrogen antagonistic effect: inhibits the proliferation of breast and endometrium.

As a PTHa that promotes bone formation, teriparatide can enhance osteoblast activity, promote bone formation, increase bone mineral density, improve bone quality, and reduce the risk of vertebral and non-vertebral fractures [7].

Representative drugs of active vitamin D and its analogues are 1α-hydroxyvitamin D_3_ (alfacalcidol) and 1,25 (OH)_2_ -VD_3_ (calcitriol). They are more suitable for the elderly, patients with osteoporosis complicated with renal insufficiency and with 1α hydroxylase deficiency or reduction, which can increase bone density, reduce falls, and the incidence of fractures [8].

As an inhibitor of nuclear factor kappa-B receptor activating factor ligand (RANKL), denosumab can inhibit the binding of RANKL to its receptor and reduce the formation, function and survival of osteoclasts, thus reducing bone resorption, increasing bone mass and improving the strength of cortical or cancellous bone [9].

The above-mentioned different types of drugs have different mechanisms of action. Generally speaking, they can be summarized as anti-bone resorption and promoting bone formation. However, there are few studies that can comprehensively compare these drugs. This article compares their efficacy and safety through a NMA, which provides more valuable suggestions for clinical medication.

## Materials and methods

This study is reported in accordance with PRISMA (Preferred Reporting Items for Systematic Reviews and Meta-Analyses) (see S1 File) and AMSTAR (Assessing the methodological quality of systematic reviews) (see S2 File).

### Search strategy and selection criteria

We searched randomized controlled trials published by PubMed, Embase and Cochrane Library until March 2020. The keywords are "Glucocorticoid(s)"or"corticoid(s)"or"corticosteroid(s)"or"tmethylprednisolone"or"prednisone"or"prednisolone"or"hydrocortisone"or"triamcinolone"or"dexamethasone" and "osteoporosis".

The inclusion criteria are as follows: (1)Patients were at least 18 years old;(2) Patients had taken prednisone or its equivalent at a dosage of ≥5 mg/day for≥3 months prior to screening; (3)Patients were required to have a LS or TH BMD T score of ≤−2.0 or ≤−1.0 plus at least one fragility fracture while taking glucocorticoids; (4)Language was English; (5) Studies were RCTs.

The exclusion criteria are as follows:(1) Primary osteoporosis (including postmenopausal osteoporosis, senile osteoporosis and idiopathic osteoporosis) and other secondary osteoporosis caused by non-glucocorticoid; (2) The type of articles was review, meta-analysis, and other non-RCT; (3) The content and outcome are not the incidence of vertebral fracture and the change of BMD.

### Data extraction and quality assessment

The main outcome that this study focuses on were the incidence of vertebral and non-vertebral fracture, and the secondary outcome were mean percent changes from baseline to at least 12 months in BMD of the FN and TH, and the incidence of serious adverse events.

In this paper, two persons independently conducted literature search, screening, data extraction and heterogeneity analysis. If there is any objection, they will reach an agreement after discussion, complete the preliminary search according to the established search strategy, and read the abstract and full text to exclude studies that do not meet the inclusion criteria.

### Data synthesis and analysis

All values are expressed as mean ± SD. We use the risk ratio (RR) and its 95% confidence interval (CI) as the effect index for discontinuous data, and the standardized mean difference (SMD) and its 95% confidence interval (CI) as the effect index of continuous data. We use the calculated estimated variance (τ^2^) to evaluate the statistical heterogeneity, use the surface under the cumulative ranking curve (SUCRA) to rank the efficacy and safety of these drugs, The larger the value, the higher the ranking. The loop-specific heterogeneity test is used to evaluate the inconsistency between direct comparison and inter-comparison. If p<0.05, it means that there is a statistically significant inconsistency. A funnel chart is drawn to detect publication bias. All data are analyzed by stata16 MP.

## Results

### Search results and characteristics of included studies

**Fig 1:**
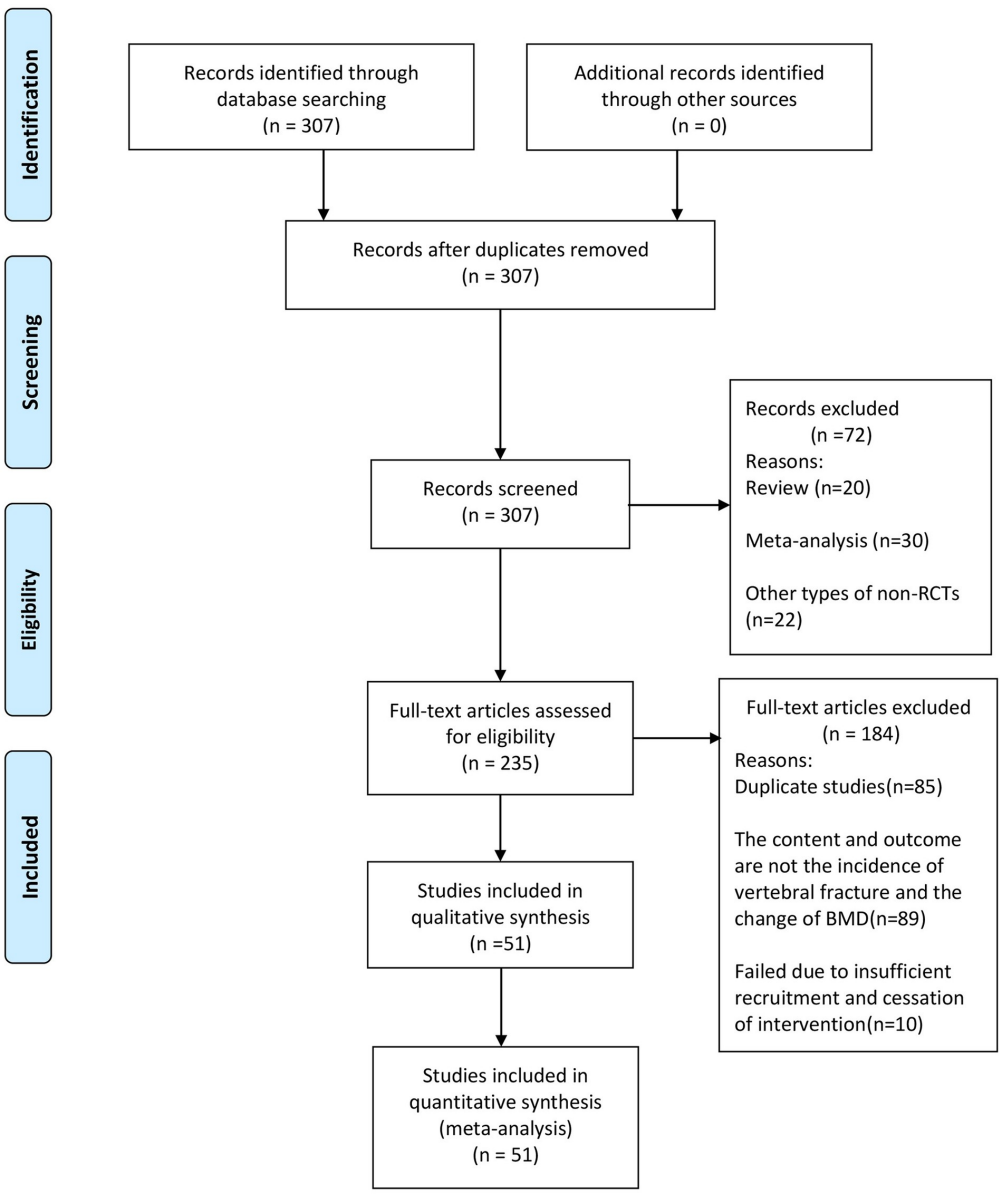
Flow diagram of literature search and study inclusion.

We searched the PubMed, Embase and Cochrane Library for studies on the treatment of GIOP. Initially, there were 307 articles, 72 of which were excluded due to non-RCTs, including 20 reviews, 30 meta-analysis, and 22 other types of non-RCTs. We screened the remaining 235 full-text studies, and excluded 184, including 85 duplicate studies. The content and outcome of 89 studies are not the incidence of vertebral fracture and the change of BMD, and 10 studies failed due to insufficient recruitment and cessation of intervention (Fig 1).

Our study included 51 randomized controlled trials [10–60], a total of 6803 subjects, a total of 18 drugs were analyzed and compared, they are alendronate, alfacalcidol, calcium, teriparatide, denosumab, calcitonin, pamidronate, zoledronic acid, risedronate, clodronate, etidronate, parathyroid hormone, raloxifene, sodium fluoride (NaF), eldecalcitol, monofluorophosphate, minodronate, ibandronate, Vitamin D_3_.

Table 1 shows the basic characteristics of these studies, including the first author and published year; the dose and duration of patients taking glucocorticoids; the patient’s age, gender, BMD or T-score of LS; and the number and proportion of menopausal women among them.

**Table 1:** Characteristics of the included studies*^a^.

Comparison	n	GC dose(mg/d)^b^	GC Duration(m)	Age(y)	Sex (M/F)	postmenopausal n (%)	LS BMD (gm/cm2) or T-score
**Ron N.J. de Nijs 2007**							
Alendronate	99	23±20	>6	60±14	40/59	52(52.5)	0.99±0.17
Alfacalcidol	101	22±18	>6	62±15	36/65	55(54.5)	1.02±0.16
**Seiji Takeda 2008**							
Alendronate	17	12.1 ± 6.6	>6	49.2±14.6	0/17	11 (64.7)	0.838 ± 0.153
Alfacalcidol	16	11.5 ± 10.5	>6	45.0±13.2	0/16	7 (43.8)	0.893 ± 0.132
**S. Kitazai 2008**^c^							
Alendronate	16	9.7 ± 9.7	110.4± 108	41.2 ± 12.8	10/6	NM	0.926 ±0.098
Alfacalcidol	20	10.9 ± 6.5	67.2± 73.2	38.1 ± 15.5	12/8	NM	0.906 ± 0.125
**S.Aubrey.Stoch 2009**							
Alendronate	114	16.5±11.6	54.6±72.0	51.9±14.4	44/70	29(25.4)	-0.33±1.37
Placebo	59	15.6±12.0	44.8±63.0	54.6±14.8	28/31	17(28.8)	0.38±1.11
**Philip N Sambrook 2002**							
Alendronate	64	12.0±9.9	>6	62.4±13.5	20/44	NM	1.02±0.20
Calcitriol	67	15.8±15.4	>6	57.9±13.0	21/46	NM	1.07±0.24
**Johannes W.G. Jacobs 2007**							
Alendronate	99	23±20	>6	60±14	40/59	52(52.5)	1.06±0.21
Alfacalcidol	101	22±18	>6	62±15	36/65	55(54.5)	1.09±0.21
**Ken Iseri 2018**							
Denosumab	14	5.0	6.9	66.5	6/8	5 (35.7)	0.895
Alendronate	14	5.0	9.0	65.5	6/8	4 (28.6)	0.875
**Funda Tascioglu 2004**							
Alendronate	22	8.00±1.77	48.00±21.12	55.67±6.67	0/22	22(100.0)	0.69±0.07
Calcitonin	24	7.58±2.04	54.48±27.36	58.13±6.51	0/24	24(100.0)	0.68±0.07
**Shegeki Yamada 2007**							
Risedronate	6	3.5±1.7	33.3±5.7	69.2±6.0	0/6	6(100)	0.64±0.10
Alfacalcidol	6	3.8±2.8	25.6±12.3	72.0±8.7	0/6	6(100)	0.64±0.10
**Jese S. Siffledeen 2005**							
Etidronate	72	NM	5.6 ±1.9	40.0±12.1	38/34	NM	0.94±0.10
Placebo	71	NM	5.4 ±1.6	40.1±14.1	34/37	NM	0.91±0.11
**Kenneth G. Saag 2007**							
alendronate	214	7.8	14.4	57.3±14.0	41/173	143 (82.7)	0.85±0.13
teriparatide	214	7.5	18	56.1±13.4	42/172	134 (77.9)	0.85±0.13
**Benito R. Losada 2008**							
alendronate	32	7.5±1.7	5.3±2.9	54.9±4.5	5/27	NM	0.8 ±0.05
teriparatide	29	8.8±1.9	2.7±3.2	52.5 ±5.0	5/24	NM	0.8 ±0.05
**Alan L. Burshell 2009**							
alendronate	77	8.0	16.8	60.6±2.5	17/60	50(64.9)	−2.7±0.1
teriparatide	80	7.5	14.4	56.1±2.6	13/67	41(51.3)	−2.5±0.1
**Jean-Pierre 2009**							
alendronate	192	10.1±0.7	5.1 ± 0.5	57.1±1.0	NM	NM	0.85±0.01
teriparatide	195	9.4±0.4	5.2 ± 0.6	55.8±1.0	NM	NM	0.85±0.01
**B. L. Langdahl 2009** **alendronate**							
Postmenopausal	143	7.3	26.4	62.1±1.2	0/143	143(100)	−2.7±0.1
Premenopausal	30	10.0	10.8	35.8±2.1	0/30	0	−2.6±0.2
Men	41	10.0	25.2	59.7±1.9	41/0	0	−2.3±0.2
**teriparatide**							
Postmenopausal	134	7	31.2	61.9±1.2	0/134	134(100)	−2.7±0.1
Premenopausal	37	8	21.6	40.0±1.9	0/37	0	−2.4±0.2
Men	42	10	27.6	55.5±1.9	42/0	0	−2.3±0.2
**Kenneth G. Saag 2009**							
alendronate	214	≥5	24	57.3±14.0	41/173	143 (66.8)	0.864±0.014
teriparatide	214	≥5	27.6	56.1±13.4	42/172	134 (62.6)	0.863±0.014
**Kenneth G Saag 2016**							
alendronate	214	7.5	48	57±14	41/173	NM	-2.5 ± 0.1
teriparatide	214	7.5	27.6	56±13	42/172	NM	-2.4 ± 0.1
**Kenneth G. Sagg 1998**							
placebo	159	10	NM	54±15	52/107	67 (42.1)	0.95±0.16
alendronate	157	10	NM	55±15	44/113	83 (52.9)	0.93±0.16
**S.Aubrey.Stoch 2009**							
Alendronate	114	16.5±11.6	54.6±72.0	51.9±14.4	44/70	29(25.4)	-0.33±1.37
Placebo	59	15.6±12.0	44.8±63.0	54.6±14.8	28/31	17(28.8)	0.38±1.11
**Jonathan D. Adachi 2000**							
Placebo	61	20.4 ± 20.7	≥3	54 ± 15	19/42	25(41.0)	0.93 ± 0.15
Alendronate	55	17.4 ± 18.0	≥3	53 ± 15	15/40	26(47.3)	0.93 ± 0.15
**Chi Chiu Mok 2010**							
Raloxifene	57	7.2±6.2	58.1	55.4±7.8	0/57	57(100)	0.864±0.136
Placebo	57	6.5±5.5	67.8	55.2±7.6	0/57	57(100)	0.848±0.147
**David M Reid 2009**							
Zoledronic acid	272	10	≥12	53.2 ±14.0	87/185	118(43.4)	–1.34±1.34
Risedronate	273	10	≥12	52.7 ±13.7	90/183	117(42.9)	–1.40±1.28
**Philip N Sambrook 2011**							
Zoledronic acid	75	15.3±13.11	>3	57.2±14.73	75/0	0	0.929±0.152
Risedronate	77	15.5±12.12	>3	55.7±13.95	77/0	0	0.920±0.139
**Claus-C.Glüer 2012**							
Teriparatide	45	8.8	85.2	57.5±12.8	NM	NM	-2.48
Risedronate	47	8.8	58.8	55.1±15.5	NM	NM	-2.33
**Kenneth G. Saag 2019**							
Risedronate	252	11.1 ± 7.69	≥3	61.3±11.1	67/185	157(62.3)	–1.96 ± 1.38
Denosumab	253	12.3 ± 8.09	≥3	61.5±11.6	68/185	159(62.8)	–1.92 ± 1.38
**R. Eastell 1999**^d^							
Placebo	40	812±286	199.2	65.0±6.3	0/40	40(100)	0.76±0.13
Risedronate	40	810±298	162	64.5±7.2	0/40	40(100)	0.80 ± 0.13
**David M. Reid 1999**							
Placebo	96	15±13	62±72	59±12	36/60	53±55.2	-1.7±1.5
Risedronate	100	15±12	57±58	58±12	36/64	55±55.0	-1.7±1.6
**Sonsoles Guadalix 2011**^d^							
Risedronate	45	3931.2±2129.4	12	57.9 ± 6.5	32/13	13(28.9)	0.792 ± 0.104
Placebo	44	4584.0±2638.6	12	54.6 ± 8.8	38/6	4(9.1)	0.844 ± 0.089
**Naohiko Fujii 2006**							
Placebo	37	10.6±5.1	6.5±8.1	42.2±16.5	16/21	6(16.2)	1.094±0.119
risedronate	40	9.9±5.0	5.2±6.3	40.0±16.3	15/25	6(15.0)	1.054±0.137
**A Rmando T Orres 2004**							
Calcitriol	45	10	12	46.7±12.2	37/8	3(6.7)	1.02 ± 0.12
Placebo	41	10	12	51.1±11.9	30/11	7(17.1)	0.98 ± 0.12
**Toshio Matsumoto 2020**							
Eldecalcitol	178	10.3±9.0	>3	58.5±16.2	62/116	72 (62.1)	− 0.70±1.39)
Alfacalcidol	182	9.5±7.7	>3	58.4±15.7	59/123	75 (61.0)	− 0.54±1.39)
**J. D. Ringe 1999**							
Alfacalcidol	43	9.7	70.8	60.6	15/28	NM	−3.28
vitamin D	42	9.6	49.2	60.7	15/27	NM	−3.25
**J. D. Ringe 2003**							
Alfacalcidol	103	8.0	36	60.1±9.8	38/65	NM	3.26±0.57
vitamin D	101	7.5	36	60.3±9.9	36/65	NM	3.25±0.39
**Satoshi Soen 2019**							
Minodronate	40	7.53 ± 6.57	44.0 ± 48.3	62.0±13.5	17/23	NM	
Placebo	42	7.62 ± 5.74	41.5 ± 42.5	61.3 ± 9.6	23/19	NM	93.1 ± 16.0
**P.Pitt 1997**							
etidronate	26	8.2±4.2	104	58.9±13.7	10/16	NM	0.74±0.12
placebo	23	7.2±4.0	104	59.2±10.8	9/14	NM	0.76±0.11
**Christian Roux 1998**							
placebo	58	≥7.5	≥12	59.0±13.6	20/38	30(51.7)	0.924±0.156
etidronate	59	≥7.5	≥12	58.5±13.9	22/37	27(45.8)	0.897±0.158
**Jacques P. Brown 2001**		22.7 ± 21.7					
placebo	61	22.7 ± 21.7	≥52	60 ± 17	24/37	29(47.5)	NM
etidronate	53	20.5 ± 22.2	≥52	64 ± 13	17/36	29(54.7)	NM
**I.Garcia-Delgado 1996**	SE						
Calcitonin	13	NM	NM	55.9±1.63	13/0	NM	0.854 ± 0.069
Etidronate	14	NM	NM	52.7±1.82	14/0	NM	0.871 ± 0.091
**Y. Boutsen 1997**							
Pamidronate	14	31.2±23.8	NM	60±16	3/11	3(21.4)	0.857±0.118
Calcium	13	28.1±23.8	NM	61±12	2/11	2(15.4)	0.960±0.161
**Y. Boutsen 2000**							
Pamidronate	9	≥10	≥3	59±21	4/5	4(44.4)	0.965±0.161
Calcium	9	≥10	≥3	57±18	4/5	4(44.4)	0.963±0.173
**T. Bianda 2000**^d^							
Calcitonin	12	14800 ± 1200	12	54.5 ± 1.0	11/1	NM	0.97 ± 0.04
Pamidronate	14	13800 ± 1700	12	51.1 ± 3.0	13/1	NM	1.01 ± 0.03
**Se Hwa Kim 2003**							
Placebo	20	NM	NM	48±18	9/11	7(35.0)	0.897±0.193
Pamidronate	25	NM	NM	49±15	14/11	7(28.0)	0.864±0.185
**A Nzeusseu Toukap 2005**							
Pamidronate	16	≥7.5	≥12	30.5±7.4	NM	NM	0.954±0.108
Placebo	14	≥7.5	≥12	25.3±9.2	NM	NM	0.974±0.147
**B. Frediani 2003**							
Clodronate	84	8.4 ± 3.2	NM	61.1±12.2	0/84	63(75.0)	0.99 ± 0.18
Placebo	79	8.9 ± 4.1	NM	62.4±13.4	0/79	61(77.2)	0.98 ± 0.16
**Vered Abitbol 2007**							
Clodronate	33	15.0	12	30	16/17	NM	-1.3±1.10
Placebo	34	14.0	12	30	14/20	NM	-1.2±1.33
**CC Mok 2013**							
Raloxifene	30	7.9±7.4	89.2±71	52.5±6.7	0/30	30(100)	0.883±0.125
Placebo	32	5.8±2.6	84.7±65	52.5±6.8	0/32	32(100)	0.886±0.134
**M Hakala 2012**							
Ibandronate	68	6.71±2.71	40±54	64±8	0/68	68(100)	1.128±0.11
Placebo	72	6.67±2.79	44±66	63±7	0/72	72(100)	1.146±0.15
**W.F.Lems 1997**							
Placebo	24	21.2±17.3	≥6	53±15	10/14	8(33.3)	1.043±0.183
NaF	20	14.6±10.5	≥6	49±17	7/13	4(20.0)	1.014±0.131
**Willem F Lems 1997**							
Placebo	24	16.9±19.8	≥6	60±17	9/15	10(41.7)	0.944±0.167
NaF	23	10.6±4.2	≥6	56±17	5/18	15(65.2)	0.804±0.142
**G.Guaydier-Souquibres 1995**							
Monofluorophosphate	15	15.9±9.4	56.4±39.6	15.9±9.4	12/3	NM	0.910±0.155
Placebo	13	20.4±16.2	88.8±90.0	20.4±16.22	9/4	NM	0.925 ±0.129
**R. Rizzoli 1994**^c^							
Monofluorophosphate	25	18.2±2.3	111.6±20.4	50.6±3.2	13/12	9(36.0)	- 1.52±0.19
Placebo	23	12.1±1.1	90.0±21.6	51.6±3.0	10/13	9(39.1)	- 1.19±0.18

*All Patients received supplements of calcium (1000 mg/d) and vitamin D (800 IU/d).

^a^If there is no special instructions, all values are mean ± SD.

^b^prednisone or equivalent.

^c^values are mean±SE.

^d^12 months cumulative dose of prednison(mean±SD).

BMD = bone mineral density.

LS = lumbar spine.

GC = glucocorticoid.

M = male.

F = female.

NM = not mentioned.

### Incidence of vertebral fractures

**Fig 2:**
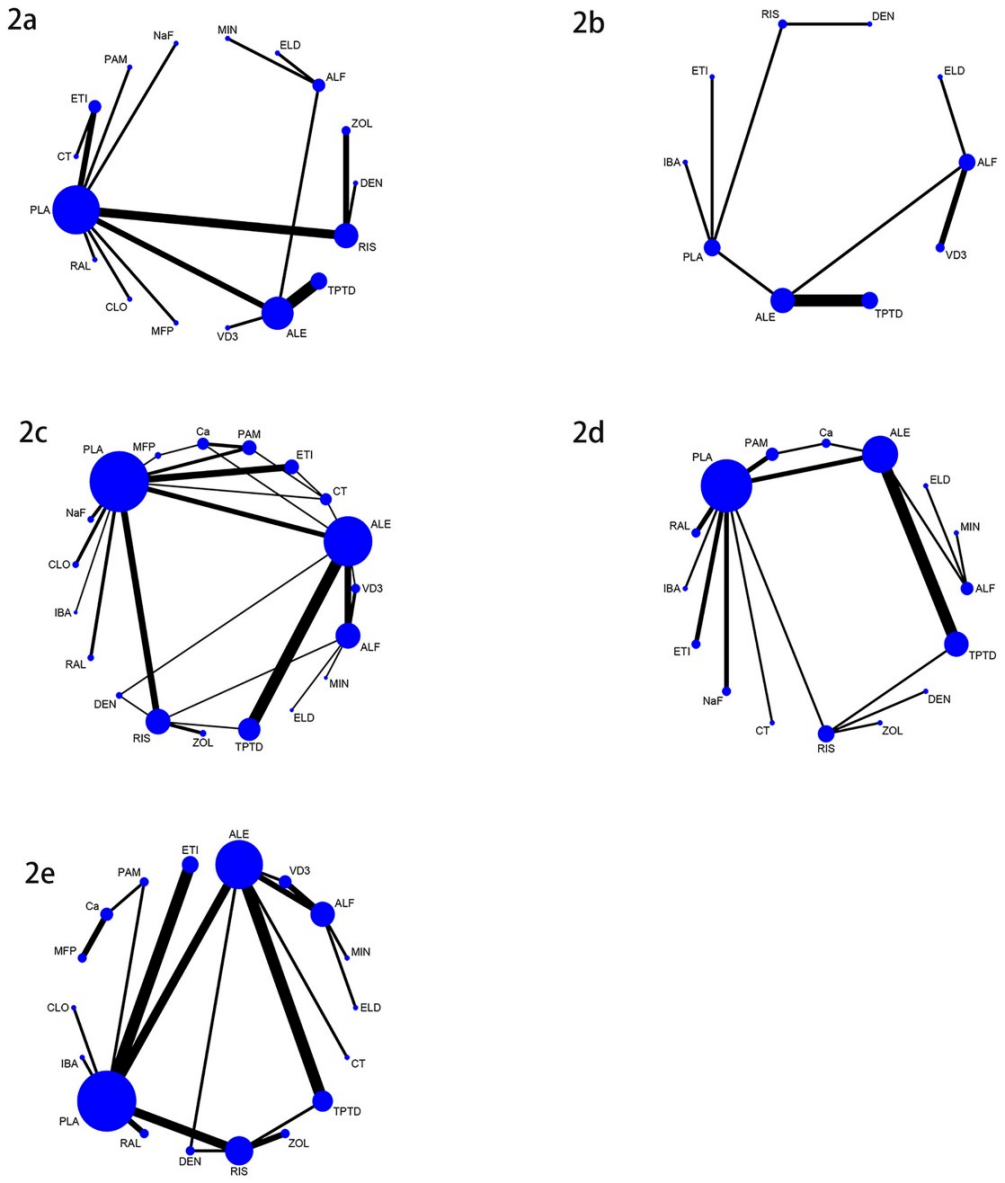
Network meta-analysis plots. (2a) Incidence of vertebral fractures, (2b) Incidence of non-vertebral fractures, (2c) Mean percentage change of BMD of LS from baseline, (2d) Mean percentage change of BMD of TH from baseline, (2e) Serious adverse events. The size of each node is positively correlated with the number of direct comparative studies of different anti-osteoporotic drugs, and the line thickness is positively correlated with the sample size included in the study.

There were 24 studies involving vertebral fractures, with a total of 4796 patients. The network relationship is shown in Fig 2a, and the included studies do not form a closed loop. It can be seen from the funnel chart that the study is uniformly distributed in the middle and upper part of the funnel, and no research falls outside the funnel diagram, so it can be considered that the risk of small sample effect or publication bias is very small (Fig 4a). In terms of reducing the incidence of vertebral fractures, teriparatide (SUCRA 95.9%) has the best effect, followed by pamidronate (SUCRA 84.3%) and raloxifene (SUCRA 78.7%), while the worst effect is minodronate (SUCRA 8.0%), the specific ranking is shown in Fig 5a and Table 2. In addition, the incidence of vertebral fractures was lower in teriparatide (RR0.06, 95%CI 0.010.27) and etidronate (RR0.29, 95%CI 0.160.51) than placebo.

**Table 2:** SUCRA ranking.

Rank or Outcomes	1	2	3	4	5	6	7	8	9	10	11	12	13	14	15	16	17	18	19
LS BMD	RAL	PAM	DEN	CLO	MFP	NaF	TPTD	Ca	CT	MIN	ALE	ELD	IBA	ALF	ZOL	ETI	RIS	PLA	VD3
TH BMD	DEN	PAM	RAL	Ca	TPTD	ALE	IBA	RIS	CT	ZOL	ETI	ELD	MIN	PLA	ALE	NaF			
VF	TPTD	PAM	RAL	ETI	VD3	CT	ALE	CLO	DEN	RIS	ELD	NaF	ZOL	PLA	ALF	MFP	MIN		
non-VF	IBA	ALE	ETI	ALF	TPTD	ELD	PLA	VD3	RIS	DEN									
AE	CT	ALF	VD3	MIN	ELD	ALE	TPTD	ETI	CLO	ZOL	RAL	Ca	DEN	MFP	PLA	RIS	PAM	IBA	

SUCRA = the surface under the cumulative ranking curve; LS = lumbar spine; TH = total hip; BMD = bone mineral density; RAL = raloxifene; PAM = pamidronate DEN = denosumab; CLO = clodronate; MFP = monofluorophosphate; NaF = sodium fluoride; TPTD = teriparatide; Ca = calcium; CT = calcitonin; MIN = minodronate; ALE = alendronate; ELD = eldecalcitol; IBA = ibandronate; ALF = alfacalcidol; ZOL = zoledronic acid; ETI = etidronate; RIS = risedronate; PLA = placebo; VD3 = Vitamin D_3_; VF = vertebral fractures; non-VF = non-vertebral fractures; AE = adverse events.

### Incidence of non-vertebral fractures

There were 13 studies on non-vertebral fractures, with a total of 3455 patients. The network relationship is shown in Fig 2b, and the included studies do not form a closed loop. From the funnel chart, we can see that the included research is not very balanced, basically distributed at the top of the funnel, and no research falls outside the funnel chart. The risk of small sample effect or publication bias is relatively high (Fig 4b). In reducing the incidence of non-vertebral fracture, ibandronate (SUCRA 75.2%) is the best, followed by alendronate (SUCRA 70.2%) and etidronate (SUCRA 67.2%), while the worst effect is denosumab (SUCRA 19.6%). The specific ranking is shown in Fig 5b and Table 2.

### Mean percentage change of BMD of LS from baseline

**Fig 3:**
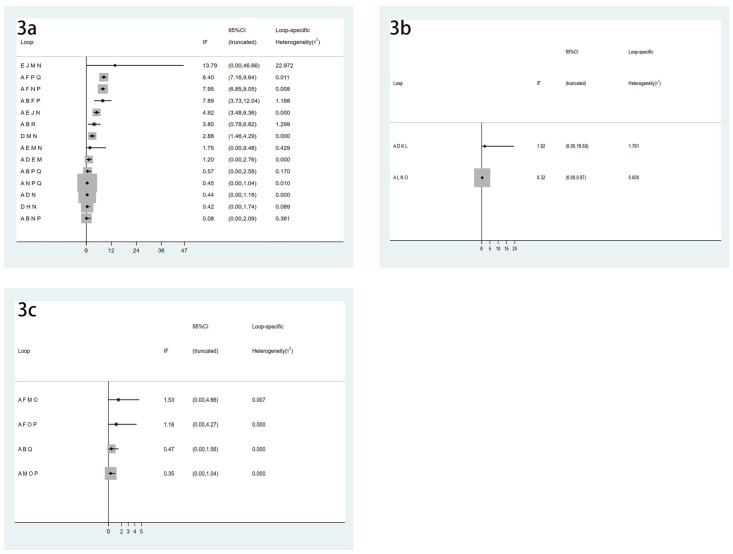
Loop-specific heterogeneity diagram. (3a) Mean percentage change of BMD of LS from baseline, (3b) Mean percentage change of BMD of TH from baseline, (3c) Serious adverse events.

**Fig 4:**
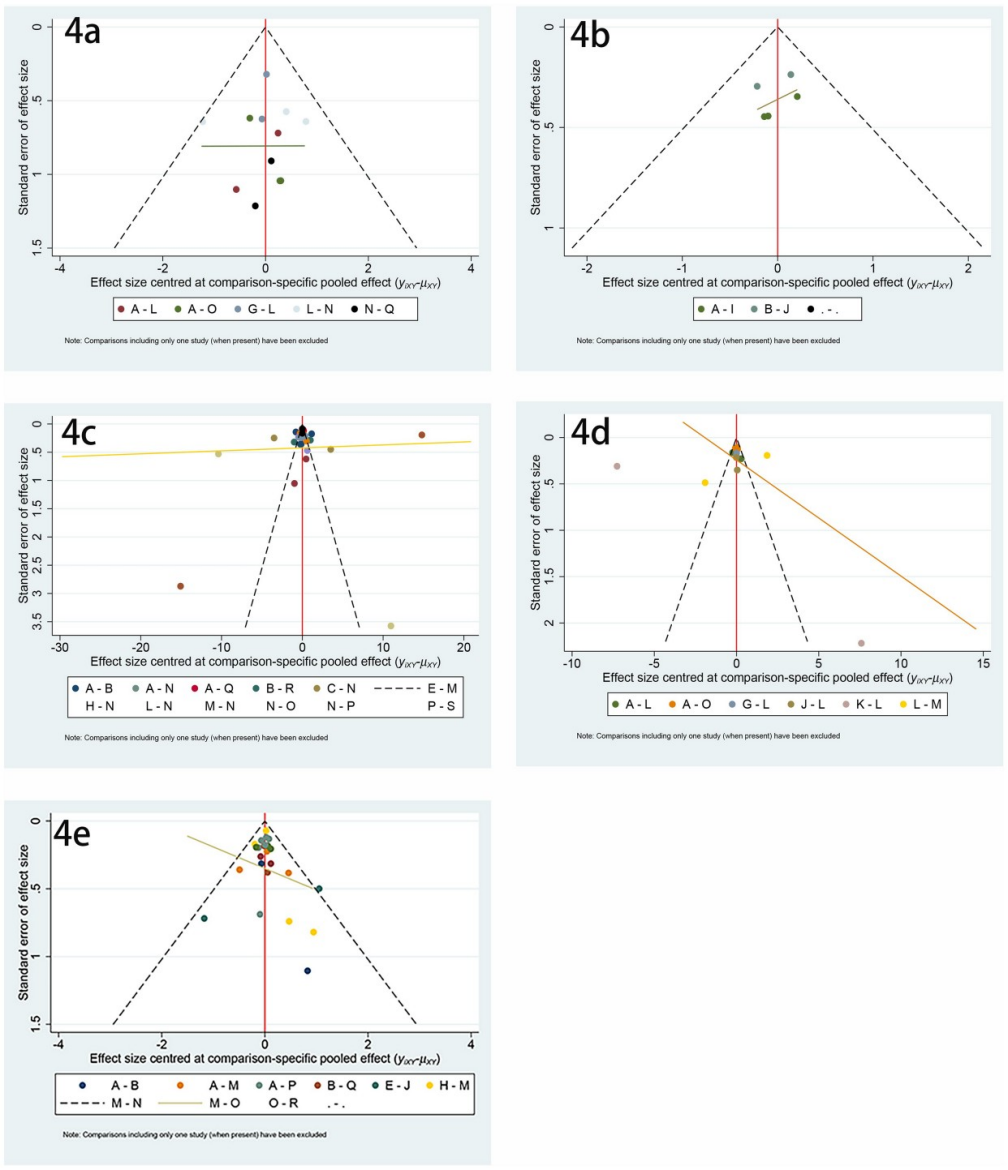
Funnel chart. (4a) Incidence of vertebral fractures, (4b) Incidence of non-vertebral fractures, (4c) Mean percentage change of BMD of LS from baseline, (4d) Mean percentage change of BMD of TH from baseline, (4e) Serious adverse events.

**Fig 5:**
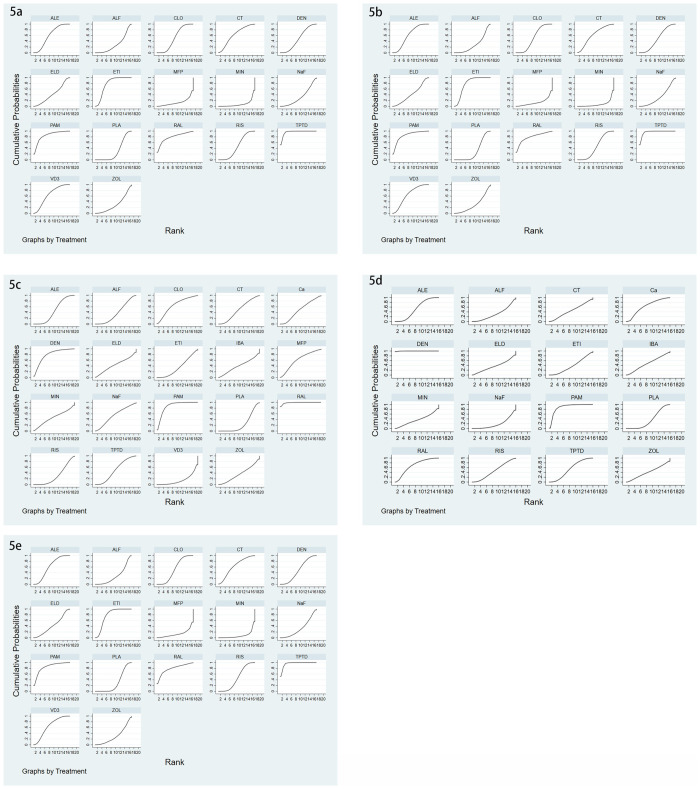
The surface under the cumulative ranking curve (SUCRA) ranking chart. (5a) Incidence of vertebral fractures, (5b) Incidence of non-vertebral fractures, (5c) Mean percentage change of BMD of LS from baseline, (5d) Mean percentage change of BMD of TH from baseline, (5e) Serious adverse events.

There were 51 articles studying the changes of BMD of LS, involving a total of 6803 subjects. The network relationship is shown in Fig 2c, the consistency test is shown in Fig 3a, and the funnel chart is shown in Fig 4c, which shows that the included studies are more symmetrical, most of the studies are at the top, but very few studies are in the lower part of the funnel and outside. Therefore, the risk of small sample effect or publication bias is small. Among various types of anti-osteoporosis drugs, raloxifene (SUCRA 98.5%) is the best in increasing LS BMD, followed by pamidronate (SUCRA 86.2%) and denosumab (SUCRA 78.9%). On the contrary, the worst effect is Vitamin D_3_ (SUCRA 15.6%), the specific ranking is shown in Fig 5c and Table 2. Moreover, compared with placebo, raloxifene (SMD12.56, 95%CI 6.33–18.78) and pamidronate (SMD 6.84, 95%CI 2.26–11.42) significantly increased LS BMD.

### Mean percentage change of BMD of TH from baseline

There were 26 studies involving changes in TH BMD, with a total of 3946 patients. The network relationship is shown in Fig 2d, and the consistency test is shown in Fig 3b. It can be seen from the funnel chart that most of the studies are at the top, but there are 4 studies outside the funnel chart, so the risk of small sample effects or publication bias is not excluded (Fig 4d). Among various types of anti-osteoporosis drugs, denosumab (SUCRA 99.7%) is the best in increasing total hip bone density, followed by pamidronate (SUCRA 87.9%) and raloxifene (SUCRA 68.5) %), and the worst effect is NaF (sodium fluoride) (SUCRA 19.1%), the specific ranking is shown in Fig 5d and Table 2. Compared with placebo, denosumab (SMD12.63, 95%CI 6.51–18.75 and pamidronate (SMD5.14, 95%CI 3.15–8.94) increased the BMD of the TH.

### Serious adverse events

There were 35 studies on adverse reactions, with a total of 6028 patients. The network relationship is shown in Fig 2e, and the consistency test is shown in Fig 3c. As can be seen from the funnel chart, the included studies are not very balanced, most of them are distributed at the top of the funnel, and one study falls outside the funnel chart, which does not rule out the risk of small sample effect or publication bias (Fig 4e). In terms of the incidence of adverse reactions, calcitonin (SUCRA 92.4%) is the best, followed by alfacalcidol (SUCRA 81.5%) and Vitamin D_3_ (SUCRA 79.3%), while the worst effect is ibandronate (SUCRA 15.5%). The specific ranking is shown in Fig 5e and Table 2.

## Discussion

We conducted a NMA of different types of anti-osteoporosis drugs and reached the following conclusions: Among the different types of anti-osteoporosis drugs, teriparatide (SUCRA 95.9%) has the best effect in reducing the incidence of vertebral fractures; ibandronate (SUCRA 75.2%) has the best effect in reducing the incidence of non-vertebral fractures; raloxifene (SUCRA 98.5%) has the best effect in increasing LS BMD; denosumab (SUCRA 99.7%) is the best in increasing TH BMD; calcitonin (SUCRA 92.4%) has the lowest incidence of adverse events.

We obtained the following results through NMA of different kinds of anti-osteoporotic drugs. Compared with placebo, the incidence of vertebral fracture was very low in teriparatide (RR0.06, 95%CI 0.01–0.27) and etidronate (RR0.29, 95%CI 0.16–0.51); raloxifene (SMD12.56, 95%CI 6.33–18.78) and pamidronate (SMD 6.84, 95%CI 2.26–11.42) significantly increased LS BMD; denosumab (SMD12.63, 95%CI 6.51–18.75) and pamidronate (SMD5.14, 95%CI 3.15–8.94) increased BMD of the TH. There were no significant differences in the incidence of nonvertebral fractures or adverse effects of the other drugs compared with placebo.

Previous NMA showed that teriparatide was the most effective anti-osteoporotic drug for vertebral fractures [61–66] and the lowest incidence of ibandronate for non-vertebral fractures [61,63]. These two conclusions are consistent with this study. For the increase of LS BMD, the results of, M. A. Amiche et al. [61] show that ibandronate is the best, while this paper found that raloxifene is the best, we should be cautious about the differences in these results.

In addition, our analysis shows that vitamin D analogues (such as calcitriol) and active metabolites (such as alfacalcidol) may be more effective in preventing fractures than vitamin D alone. This provides an evidence-based medicine basis for clinical drug use in the future. Vitamin D should not be used only, but its analogues and active metabolites should be used in combination.

Although the efficacy of the above anti-osteoporotic drugs is significant, their adverse reactions cannot be ignored at the same time. As one of the representative drugs of bisphosphate, the main adverse events of ibandronate are gastrointestinal reactions, including epigastric pain, acid regurgitation, inflammation of the esophagus and stomach and so on. Other adverse reactions include affecting renal function, so patients with GFR less than 35 mL/min should disable ibandronate. In addition, the lower incidence of adverse events included osteonecrosis of the jaw and atypical femur fracture [67]. A randomized controlled trial showed that adverse events to teriparatide included nausea (18%), headaches (13%) and leg cramps (3%) [7]. The main adverse reactions of denosumab are infections, such as urinary tract infection, sinusitis, pharyngitis, bronchitis and cellulitis. Others include joint pain and hypocalcemia [68]. Raloxifene is well tolerated, the side effects are limited to hot flashes and vaginal dryness, and the risk of thromboembolism is slightly increased [69]. Intranasal calcitonin can cause rhinitis, nosebleeds and allergic reactions, especially in people with a history of salmon allergy [70].

This article has the following advantages. First, this article is to study the most complete mesh meta-analysis of anti-osteoporosis drugs. Second, this article is an earlier study of an NMA of anti-osteoporosis drugs on the BMD of the LS and TH. Third, this article first includes several drugs that have not been studied in previous NMA, including calcitonin, clodronate, sodium fluoride, eldecalcitol, monofluorophosphate, and mineralronate.

However, there are some shortcomings in our research. First, the menopause of female subjects may affect the efficacy of the drug. Second, the patients included in this study were given long-term calcium and vitamin D supplementation, which also had an impact on the efficacy of the drug. Third, the research time of the articles included in this paper varies greatly, from 12 months to 36 months, or even longer. Fourth, the number of randomized controlled trials for direct comparison of some drugs included in this paper is relatively small, which leads to the fact that the results of indirect comparison may not be very persuasive and should be treated with caution. Last, this paper includes the original research of different countries and regions, which is also one of the limitations of this paper. Therefore, more experiments are needed to verify or correct the results of this paper.

## Conclusion

In terms of the incidence of vertebral and non-vertebral fractures, teriparatide and ibandronate are the most effective drugs. Raloxifene and denosumab have the most significant effect on increasing BMD of LS and TH. There was no significant difference in the incidence of adverse events among different drugs.

## Supporting information

S1 Table Characteristics of the included studies. (DOCX)

S2 Table SUCRA ranking. (DOCX)

S1 File The PRISMA network meta-analysis checklist. (DOCX)

S2 File Risk of bias summary: Review authors’ judgements about each risk of bias item for each included study. (PNG)

S3 File Risk of bias graph: Review authors’ judgements about each risk of bias item presented as percentages across all included studies. (PNG)

S4 File Search strategy. (DOCX)

S5 File Risk of bias summary. (DOCX)

S6 File Minimal data set. (XLSX)

## References

[1] Chiodini I, Falchetti A, Merlotti D, Eller Vainicher C, Gennari L. Updates in epidemiology, pathophysiology and management strategies of glucocorticoid-induced osteoporosis. Expert Rev Endocrinol Metab. 2020 7;15(4):283–298. 10.1080/17446651.2020.1772051. . 32584619

[2] Mazziotti G, Giustina A, Canalis E, Bilezikian JP. Glucocorticoid-induced osteoporosis: clinical and therapeutic aspects. Arq Bras Endocrinol Metabol. 2007 11;51(8):1404–12. 10.1590/s0004-27302007000800028. . 18209880

[3] Amiche MA, Albaum JM, Tadrous M, Pechlivanoglou P, Lévesque LE, Adachi JD, et al Fracture risk in oral glucocorticoid users: a Bayesian meta-regression leveraging control arms of osteoporosis clinical trials. Osteoporos Int. 2016 5;27(5):1709–18. 10.1007/s00198-015-3455-9. . 26694595

[4] Hu K, Adachi JD. Glucocorticoid induced osteoporosis. Expert Rev Endocrinol Metab. 2019 7;14(4):259–266. 10.1080/17446651.2019.1617131. . 31094232

[5] Chiodini I, Merlotti D, Falchetti A, Gennari L. Treatment options for glucocorticoid-induced osteoporosis. Expert Opin Pharmacother. 2020 4;21(6):721–732. 10.1080/14656566.2020.1721467. . 32004105

[6] Chesnut CH 3rd, Azria M, Silverman S, Engelhardt M, Olson M, Mindeholm L. Salmon calcitonin: a review of current and future therapeutic indications. Osteoporos Int. 2008 4;19(4):479–91. 10.1007/s00198-007-0490-1. . 18071651

[7] Neer RM, Arnaud CD, Zanchetta JR, Prince R, Gaich GA, Reginster JY, et al Effect of parathyroid hormone (1–34) on fractures and bone mineral density in postmenopausal women with osteoporosis. N Engl J Med. 2001 5 10;344(19):1434–41. 10.1056/NEJM200105103441904. . 11346808

[8] Bischoff-Ferrari HA, Dawson-Hughes B, Staehelin HB, Orav JE, Stuck AE, Theiler R, et al Fall prevention with supplemental and active forms of vitamin D: a meta-analysis of randomised controlled trials. BMJ. 2009 10 1;339:b3692 10.1136/bmj.b3692. . 19797342 PMC2755728

[9] Yamaguchi Y, Morita T, Kumanogoh A. The therapeutic efficacy of denosumab for the loss of bone mineral density in glucocorticoid-induced osteoporosis: a meta-analysis. Rheumatol Adv Pract. 2020 3 13;4(1):rkaa008 10.1093/rap/rkaa008. . 32373775 PMC7197806

[10] Yeap SS, Fauzi AR, Kong NC, Halim AG, Soehardy Z, Rahimah I, et al A comparison of calcium, calcitriol, and alendronate in corticosteroid-treated premenopausal patients with systemic lupus erythematosus. J Rheumatol. 2008 12;35(12):2344–7. 10.3899/jrheum.080634. . 19004038

[11] Losada BR, Zanchetta JR, Zerbini C, Molina JF, De la Peña P, Liu CC, et al Active comparator trial of teriparatide vs alendronate for treating glucocorticoid-induced osteoporosis: results from the Hispanic and non-Hispanic cohorts. J Clin Densitom. 2009 Jan-Mar;12(1):63–70. 10.1016/j.jocd.2008.10.002. Epub 2008 Nov 22. . 19028124

[12] Saag KG, Emkey R, Schnitzer TJ, Brown JP, Hawkins F, Goemaere S, et al Alendronate for the prevention and treatment of glucocorticoid-induced osteoporosis. Glucocorticoid-Induced Osteoporosis Intervention Study Group. N Engl J Med. 1998 7 30;339(5):292–9. 10.1056/NEJM199807303390502. . 9682041

[13] de Nijs RN, Jacobs JW, Lems WF, Laan RF, Algra A, Huisman AM, et al Alendronate or alfacalcidol in glucocorticoid-induced osteoporosis. N Engl J Med. 2006 8 17;355(7):675–84. 10.1056/NEJMoa053569. . 16914703

[14] Kitazaki S, Mitsuyama K, Masuda J, Harada K, Yamasaki H, Kuwaki K, et al Clinical trial: comparison of alendronate and alfacalcidol in glucocorticoid-associated osteoporosis in patients with ulcerative colitis. Aliment Pharmacol Ther. 2009 2 15;29(4):424–30. 10.1111/j.1365-2036.2008.03899.x. . 19035979

[15] Burshell AL, Möricke R, Correa-Rotter R, Chen P, Warner MR, Dalsky GP, et al Correlations between biochemical markers of bone turnover and bone density responses in patients with glucocorticoid-induced osteoporosis treated with teriparatide or alendronate. Bone. 2010 4;46(4):935–9. 10.1016/j.bone.2009.12.032. . 20060081

[16] Takeda S, Kaneoka H, Saito T. Effect of alendronate on glucocorticoid-induced osteoporosis in Japanese women with systemic autoimmune diseases: versus alfacalcidol. Mod Rheumatol. 2008;18(3):271–6. 10.1007/s10165-008-0055-y. . 18427724

[17] Saag KG, Zanchetta JR, Devogelaer JP, Adler RA, Eastell R, See K, et al Effects of teriparatide versus alendronate for treating glucocorticoid-induced osteoporosis: thirty-six-month results of a randomized, double-blind, controlled trial. Arthritis Rheum. 2009 11;60(11):3346–55. 10.1002/art.24879. . 19877063

[18] Stoch SA, Saag KG, Greenwald M, Sebba AI, Cohen S, Verbruggen N, et al Once-weekly oral alendronate 70 mg in patients with glucocorticoid-induced bone loss: a 12-month randomized, placebo-controlled clinical trial. J Rheumatol. 2009 8;36(8):1705–14. 10.3899/jrheum.081207. . 19487264

[19] Sambrook PN, Kotowicz M, Nash P, Styles CB, Naganathan V, Henderson-Briffa KN, et al Prevention and treatment of glucocorticoid-induced osteoporosis: a comparison of calcitriol, vitamin D plus calcium, and alendronate plus calcium. J Bone Miner Res. 2003 5;18(5):919–24. 10.1359/jbmr.2003.18.5.919. . 12733733

[20] Jacobs JW, de Nijs RN, Lems WF, Geusens PP, Laan RF, Huisman AM, et al Prevention of glucocorticoid induced osteoporosis with alendronate or alfacalcidol: relations of change in bone mineral density, bone markers, and calcium homeostasis. J Rheumatol. 2007 5;34(5):1051–7. Epub 2007 Apr 1. . 17407214

[21] Saag KG, Shane E, Boonen S, Marín F, Donley DW, Taylor KA, et al Teriparatide or alendronate in glucocorticoid-induced osteoporosis. N Engl J Med. 2007 11 15;357(20):2028–39. 10.1056/NEJMoa071408. . 18003959

[22] Langdahl BL, Marin F, Shane E, Dobnig H, Zanchetta JR, Maricic M, et al Teriparatide versus alendronate for treating glucocorticoid-induced osteoporosis: an analysis by gender and menopausal status. Osteoporos Int. 2009 12;20(12):2095–104. 10.1007/s00198-009-0917-y. . 19350340

[23] Iseri K, Iyoda M, Watanabe M, Matsumoto K, Sanada D, Inoue T, et al The effects of denosumab and alendronate on glucocorticoid-induced osteoporosis in patients with glomerular disease: A randomized, controlled trial. PLoS One. 2018 3 15;13(3):e0193846 10.1371/journal.pone.0193846. . 29543887 PMC5854344

[24] Tascioglu F, Colak O, Armagan O, Alatas O, Oner C. The treatment of osteoporosis in patients with rheumatoid arthritis receiving glucocorticoids: a comparison of alendronate and intranasal salmon calcitonin. Rheumatol Int. 2005 11;26(1):21–9. 10.1007/s00296-004-0496-3. . 15688191

[25] Adachi JD, Saag KG, Delmas PD, Liberman UA, Emkey RD, Seeman E, et al Two-year effects of alendronate on bone mineral density and vertebral fracture in patients receiving glucocorticoids: a randomized, double-blind, placebo-controlled extension trial. Arthritis Rheum. 2001 1;44(1):202–11. 10.1002/1529-0131(200101)44. . 11212161

[26] Boutsen Y, Jamart J, Esselinckx W, Stoffel M, Devogelaer JP. Primary prevention of glucocorticoid-induced osteoporosis with intermittent intravenous pamidronate: a randomized trial. Calcif Tissue Int. 1997 10;61(4):266–71. 10.1007/s002239900334. . 9312195

[27] Sambrook PN, Roux C, Devogelaer JP, Saag K, Lau CS, Reginster JY, et al Bisphosphonates and glucocorticoid osteoporosis in men: results of a randomized controlled trial comparing zoledronic acid with risedronate. Bone. 2012 1;50(1):289–95. 10.1016/j.bone.2011.10.024. . 22061864

[28] Yamada S, Takagi H, Tsuchiya H, Nakajima T, Ochiai H, Ichimura A, et al Comparative studies on effect of risedronate and alfacalcidol against glucocorticoid-induced osteoporosis in rheumatoid arthritic patients. Yakugaku Zasshi. 2007 9;127(9):1491–6. 10.1248/yakushi.127.1491. . 17827929

[29] Glüer CC, Marin F, Ringe JD, Hawkins F, Möricke R, Papaioannu N, et al Comparative effects of teriparatide and risedronate in glucocorticoid-induced osteoporosis in men: 18-month results of the EuroGIOPs trial. J Bone Miner Res. 2013 6;28(6):1355–68. 10.1002/jbmr.1870. . 23322362 PMC3708101

[30] Saag KG, Pannacciulli N, Geusens P, Adachi JD, Messina OD, Morales-Torres J, et al Denosumab Versus Risedronate in Glucocorticoid-Induced Osteoporosis: Final Results of a Twenty-Four-Month Randomized, Double-Blind, Double-Dummy Trial. Arthritis Rheumatol. 2019 7;71(7):1174–1184. 10.1002/art.40874. . 30816640 PMC6619388

[31] Guadalix S, Martínez-Díaz-Guerra G, Lora D, Vargas C, Gómez-Juaristi M, Cobaleda B, et al Effect of early risedronate treatment on bone mineral density and bone turnover markers after liver transplantation: a prospective single-center study. Transpl Int. 2011 7;24(7):657–65. 10.1111/j.1432-2277.2011.01253.x. . 21466595

[32] Reid DM, Hughes RA, Laan RF, Sacco-Gibson NA, Wenderoth DH, Adami S, et al Efficacy and safety of daily risedronate in the treatment of corticosteroid-induced osteoporosis in men and women: a randomized trial. European Corticosteroid-Induced Osteoporosis Treatment Study. J Bone Miner Res. 2000 6;15(6):1006–13. 10.1359/jbmr.2000.15.6.1006. . 10841169

[33] Eastell R, Devogelaer JP, Peel NF, Chines AA, Bax DE, Sacco-Gibson N, et al Prevention of bone loss with risedronate in glucocorticoid-treated rheumatoid arthritis patients. Osteoporos Int. 2000;11(4):331–7. 10.1007/s001980070122. . 10928223

[34] Fujii N, Hamano T, Mikami S, Nagasawa Y, Isaka Y, Moriyama T, et al Risedronate, an effective treatment for glucocorticoid-induced bone loss in CKD patients with or without concomitant active vitamin D (PRIUS-CKD). Nephrol Dial Transplant. 2007 6;22(6):1601–7. 10.1093/ndt/gfl567. . 17124283

[35] Reid DM, Devogelaer JP, Saag K, Roux C, Lau CS, Reginster JY, et al Zoledronic acid and risedronate in the prevention and treatment of glucocorticoid-induced osteoporosis (HORIZON): a multicentre, double-blind, double-dummy, randomised controlled trial. Lancet. 2009 4 11;373(9671):1253–63. 10.1016/S0140-6736(09)60250-6. . 19362675

[36] Pitt P, Li F, Todd P, Webber D, Pack S, Moniz C. A double blind placebo controlled study to determine the effects of intermittent cyclical etidronate on bone mineral density in patients on long-term oral corticosteroid treatment. Thorax. 1998 5;53(5):351–6. 10.1136/thx.53.5.351. . 9708225 PMC1745232

[37] Abitbol V, Briot K, Roux C, Roy C, Seksik P, Charachon A, et al A double-blind placebo-controlled study of intravenous clodronate for prevention of steroid-induced bone loss in inflammatory bowel disease. Clin Gastroenterol Hepatol. 2007 10;5(10):1184–9. 10.1016/j.cgh.2007.05.016. Epub 2007 Aug 1. . 17683996

[38] Garcia-Delgado I, Prieto S, Gil-Fraguas L, Robles E, Rufilanchas JJ, Hawkins F. Calcitonin, etidronate, and calcidiol treatment in bone loss after cardiac transplantation. Calcif Tissue Int. 1997 2;60(2):155–9. 10.1007/s002239900206. . 9056163

[39] Mok CC, Ying SK, Ma KM, Wong CK. Effect of raloxifene on disease activity and vascular biomarkers in patients with systemic lupus erythematosus: subgroup analysis of a double-blind randomized controlled trial. Lupus. 2013 12;22(14):1470–8. 10.1177/0961203313507987. . 24113197

[40] Lems WF, Jacobs WG, Bijlsma JW, Croone A, Haanen HC, Houben HH, et al Effect of sodium fluoride on the prevention of corticosteroid-induced osteoporosis. Osteoporos Int. 1997;7(6):575–82. 10.1007/BF02652565. . 9604055

[41] Kim SH, Lim SK, Hahn JS. Effect of pamidronate on new vertebral fractures and bone mineral density in patients with malignant lymphoma receiving chemotherapy. Am J Med. 2004 4 15;116(8):524–8. 10.1016/j.amjmed.2003.12.019. . 15063813

[42] Matsumoto T, Yamamoto K, Takeuchi T, Tanaka Y, Tanaka S, Nakano T, et al Eldecalcitol is superior to alfacalcidol in maintaining bone mineral density in glucocorticoid-induced osteoporosis patients (e-GLORIA). J Bone Miner Metab. 2020 7;38(4):522–532. 10.1007/s00774-020-01091-4. . 32140784

[43] Guaydier-Souquières G, Kotzki PO, Sabatier JP, Basse-Cathalinat B, Loeb G. In corticosteroid-treated respiratory diseases, monofluorophosphate increases lumbar bone density: a double-masked randomized study. Osteoporos Int. 1996;6(2):171–7. 10.1007/BF01623943. . 8704358

[44] Lems WF, Jacobs JW, Bijlsma JW, van Veen GJ, Houben HH, Haanen HC, et al Is addition of sodium fluoride to cyclical etidronate beneficial in the treatment of corticosteroid induced osteoporosis? Ann Rheum Dis. 1997 6;56(6):357–63. 10.1136/ard.56.6.357. . 9227164 PMC1752400

[45] Soen S, Yamamoto K, Takeuchi T, Tanaka Y, Tanaka S, Ito M, et al Minodronate combined with alfacalcidol versus alfacalcidol alone for glucocorticoid-induced osteoporosis: a multicenter, randomized, comparative study. J Bone Miner Metab. 2020 7;38(4):511–521. 10.1007/s00774-019-01077-x. . 31970477

[46] Hakala M, Kröger H, Valleala H, Hienonen-Kempas T, Lehtonen-Veromaa M, Heikkinen J, et al Once-monthly oral ibandronate provides significant improvement in bone mineral density in postmenopausal women treated with glucocorticoids for inflammatory rheumatic diseases: a 12-month, randomized, double-blind, placebo-controlled trial. Scand J Rheumatol. 2012 8;41(4):260–6. 10.3109/03009742.2012.664647. . 22803768

[47] Nzeusseu Toukap A, Depresseux G, Devogelaer JP, Houssiau FA. Oral pamidronate prevents high-dose glucocorticoid-induced lumbar spine bone loss in premenopausal connective tissue disease (mainly lupus) patients. Lupus. 2005;14(7):517–20. 10.1191/0961203305lu2149oa. . 16130506

[48] Brown JP, Olszynski WP, Hodsman A, Bensen WG, Tenenhouse A, Anastassiades TP, et al Positive effect of etidronate therapy is maintained after drug is terminated in patients using corticosteroids. J Clin Densitom. 2001 Winter;4(4):363–71. 10.1385/jcd:4:4:363. . 11748341

[49] Bianda T, Linka A, Junga G, Brunner H, Steinert H, Kiowski W, et al Prevention of osteoporosis in heart transplant recipients: a comparison of calcitriol with calcitonin and pamidronate. Calcif Tissue Int. 2000 8;67(2):116–21. 10.1007/s00223001126. . 10920215

[50] Boutsen Y, Jamart J, Esselinckx W, Devogelaer JP. Primary prevention of glucocorticoid-induced osteoporosis with intravenous pamidronate and calcium: a prospective controlled 1-year study comparing a single infusion, an infusion given once every 3 months, and calcium alone. J Bone Miner Res. 2001 1;16(1):104–12. 10.1359/jbmr.2001.16.1.104. . 11149473

[51] Mok CC, Ying KY, To CH, Ho LY, Yu KL, Lee HK, et al Raloxifene for prevention of glucocorticoid-induced bone loss: a 12-month randomised double-blinded placebo-controlled trial. Ann Rheum Dis. 2011 5;70(5):778–84. 10.1136/ard.2010.143453. . 21187295

[52] Roux C, Oriente P, Laan R, Hughes RA, Ittner J, Goemaere S, et al Randomized trial of effect of cyclical etidronate in the prevention of corticosteroid-induced bone loss. Ciblos Study Group. J Clin Endocrinol Metab. 1998 4;83(4):1128–33. 10.1210/jcem.83.4.4742. . 9543129

[53] Siffledeen JS, Fedorak RN, Siminoski K, Jen H, Vaudan E, Abraham N, et al Randomized trial of etidronate plus calcium and vitamin D for treatment of low bone mineral density in Crohn’s disease. Clin Gastroenterol Hepatol. 2005 2;3(2):122–32. 10.1016/s1542-3565(04)00663-9. . 15704046

[54] Rizzoli R, Chevalley T, Slosman DO, Bonjour JP. Sodium monofluorophosphate increases vertebral bone mineral density in patients with corticosteroid-induced osteoporosis. Osteoporos Int. 1995 1;5(1):39–46. 10.1007/BF01623657. . 7703623

[55] Ringe JD, Dorst A, Faber H, Schacht E, Rahlfs VW. Superiority of alfacalcidol over plain vitamin D in the treatment of glucocorticoid-induced osteoporosis. Rheumatol Int. 2004 3;24(2):63–70. 10.1007/s00296-003-0361-9. . 14513268

[56] Ringe JD, Cöster A, Meng T, Schacht E, Umbach R. Treatment of glucocorticoid-induced osteoporosis with alfacalcidol/calcium versus vitamin D/calcium. Calcif Tissue Int. 1999 10;65(4):337–40. 10.1007/s002239900708. . 10485988

[57] Torres A, García S, Gómez A, González A, Barrios Y, Concepción MT, et al Treatment with intermittent calcitriol and calcium reduces bone loss after renal transplantation. Kidney Int. 2004 2;65(2):705–12. 10.1111/j.1523-1755.2004.00432.x. . 14717945

[58] Saag KG, Agnusdei D, Hans D, Kohlmeier LA, Krohn KD, Leib ES, et al Trabecular Bone Score in Patients With Chronic Glucocorticoid Therapy-Induced Osteoporosis Treated With Alendronate or Teriparatide. Arthritis Rheumatol. 2016 9;68(9):2122–8. 10.1002/art.39726. . 27111239

[59] Devogelaer JP, Adler RA, Recknor C, See K, Warner MR, Wong M, et al Baseline glucocorticoid dose and bone mineral density response with teriparatide or alendronate therapy in patients with glucocorticoid-induced osteoporosis. J Rheumatol. 2010 1;37(1):141–8. 10.3899/jrheum.090411. . 19918047

[60] Lane NE, Sanchez S, Modin GW, Genant HK, Pierini E, Arnaud CD. Parathyroid hormone treatment can reverse corticosteroid-induced osteoporosis. Results of a randomized controlled clinical trial. J Clin Invest. 1998 10 15;102(8):1627–33. 10.1172/JCI3914. . 9788977 PMC509014

[61] Amiche MA, Albaum JM, Tadrous M, Pechlivanoglou P, Lévesque LE, Adachi JD, et al Efficacy of osteoporosis pharmacotherapies in preventing fracture among oral glucocorticoid users: a network meta-analysis. Osteoporos Int. 2016 6;27(6):1989–98. 10.1007/s00198-015-3476-4. . 26782683

[62] Ding L, Hu J, Wang D, Liu Q, Mo Y, Tan X, et al Efficacy and Safety of First- and Second-Line Drugs to Prevent Glucocorticoid-Induced Fractures. J Clin Endocrinol Metab. 2020 1 1;105(1):dgz023 10.1210/clinem/dgz023. . 31513250

[63] Deng J, Silver Z, Huang E, Zheng E, Kavanagh K, Wen A, et al Pharmacological prevention of fractures in patients undergoing glucocorticoid therapies: a systematic review and network meta-analysis. Rheumatology (Oxford). 2020 6 23:keaa228 10.1093/rheumatology/keaa228. . 32572480

[64] Reginster J-, Bianic F, Campbell R, Martin M, Williams SA, Fitzpatrick LA. Abaloparatide for risk reduction of nonvertebral and vertebral fractures in postmenopausal women with osteoporosis: a network meta-analysis. Osteoporos Int. 2019 7;30(7):1465–1473. 10.1007/s00198-019-04947-2. . 30953114 PMC6614166

[65] Tan X, Wen F, Yang W, Xie JY, Ding LL, Mo YX. Comparative efficacy and safety of pharmacological interventions for osteoporosis in postmenopausal women: a network meta-analysis (Chongqing, China). Menopause. 2019 8;26(8):929–939. 10.1097/GME.0000000000001321. . 31021904

[66] Wang YK, Zhang YM, Qin SQ, Wang X, Ma T, Guo JB, et al Effects of alendronate for treatment of glucocorticoid-induced osteoporosis: A meta-analysis of randomized controlled trials. Medicine (Baltimore). 2018 10;97(42):e12691 10.1097/MD.0000000000012691. . 30334952 PMC6211897

[67] Cosman F, de Beur SJ, LeBoff MS, Lewiecki EM, Tanner B, Randall S, et al National Osteoporosis Foundation. Clinician’s Guide to Prevention and Treatment of Osteoporosis. Osteoporos Int. 2014 10;25(10):2359–81. 10.1007/s00198-014-2794-2. . 25182228 PMC4176573

[68] Diédhiou D, Cuny T, Sarr A, Norou Diop S, Klein M, Weryha G. Efficacy and safety of denosumab for the treatment of osteoporosis: A systematic review. Ann Endocrinol (Paris). 2015 12;76(6):650–7. 10.1016/j.ando.2015.10.009. . 26639186

[69] Delmas PD, Ensrud KE, Adachi JD, Harper KD, Sarkar S, Gennari C, et al Mulitple Outcomes of Raloxifene Evaluation Investigators. Efficacy of raloxifene on vertebral fracture risk reduction in postmenopausal women with osteoporosis: four-year results from a randomized clinical trial. J Clin Endocrinol Metab. 2002 8;87(8):3609–17. 10.1210/jcem.87.8.8750. . 12161484

[70] Cosman F, de Beur SJ, LeBoff MS, Lewiecki EM, Tanner B, Randall S, et al National Osteoporosis Foundation. Clinician’s Guide to Prevention and Treatment of Osteoporosis. Osteoporos Int. 2014 10;25(10):2359–81. 10.1007/s00198-014-2794-2. . 25182228 PMC4176573

