# Supplementary material for: Efficacy and safety of 18 anti-osteoporotic drugs in the treatment of patients with osteoporosis caused by glucocorticoid: A network meta-analysis of randomized controlled trials
Source: PLoS One. 2020 Dec 16;15(12):e0243851. doi: 10.1371/journal.pone.0243851 (PMC7743932; doi:10.1371/journal.pone.0243851)
Supplement: S1 Table — (DOCX) [file pone.0243851.s001.docx]

Table 1 Characteristics of the included studies^*a^

| Comparison | | n | | GC dose(mg/d)^b^ | | GC Duration(m) | | Age(y) | | Sex (M/F) | | postmenopausal n (%) | | LS BMD (gm/cm2) or T-score | |
| --- | --- | --- | --- | --- | --- | --- | --- | --- | --- | --- | --- | --- | --- | --- | --- |
| **RonN.J. de Nijs 2007** | |  | |  | |  | |  | |  | |  | |  | |
| Alendronate | | 99 | | 23±20 | | ＞6 | | 60±14 | | 40/59 | | 52（52.5） | | 0.99±0.17 | |
| Alfacalcidol | | 101 | | 22±18 | | ＞6 | | 62±15 | | 36/65 | | 55（54.5） | | 1.02±0.16 | |
| **Seiji Takeda 2008** | |  | |  | |  | |  | |  | |  | |  | |
| Alendronate | | 17 | | 12.1 ± 6.6 | | ＞6 | | 49.2±14.6 | | 0/17 | | 11 (64.7) | | 0.838 ± 0.153 | |
| Alfacalcidol | | 16 | | 11.5 ± 10.5 | | ＞6 | | 45.0±13.2 | | 0/16 | | 7 (43.8) | | 0.893 ± 0.132 | |
| **S. Kitazai 2008^c^** | |  | |  | |  | |  | |  | |  | |  | |
| Alendronate | | 16 | | 9.7 ± 9.7 | | 110.4± 108 | | 41.2 ± 12.8 | | 10/6 | | NM | | 0.926 ±0.098 | |
| Alfacalcidol | | 20 | | 10.9 ± 6.5 | | 67.2± 73.2 | | 38.1 ± 15.5 | | 12/8 | | NM | | 0.906 ± 0.125 | |
| **S.Aubrey.Stoch 2009** | |  | |  | |  | |  | |  | |  | |  | |
| Alendronate | | 114 | | 16.5±11.6 | | 54.6±72.0 | | 51.9±14.4 | | 44/70 | | 29(25.4) | | -0.33±1.37 | |
| Placebo | | 59 | | 15.6±12.0 | | 44.8±63.0 | | 54.6±14.8 | | 28/31 | | 17(28.8) | | 0.38±1.11 | |
| **Philip N Sambrook 2002** | |  | |  | |  | |  | |  | |  | |  | |
| Alendronate | | 64 | | 12.0±9.9 | | ＞6 | | 62.4±13.5 | | 20/44 | | NM | | 1.02±0.20 | |
| Calcitriol | | 67 | | 15.8±15.4 | | ＞6 | | 57.9±13.0 | | 21/46 | | NM | | 1.07±0.24 | |
| **Johannes　W.G. Jacobs　2007** | |  | |  | |  | |  | |  | |  | |  | |
| Alendronate | | 99 | | 23±20 | | ＞6 | | 60±14 | | 40/59 | | 52（52.5） | | 1.06±0.21 | |
| Alfacalcidol | | 101 | | 22±18 | | ＞6 | | 62±15 | | 36/65 | | 55（54.5） | | 1.09±0.21 | |
| **Ken Iseri 2018** | |  | |  | |  | |  | |  | |  | |  | |
| Denosumab | | 14 | | 5.0 | | 6.9 | | 66.5 | | 6/8 | | 5 (35.7) | | 0.895 | |
| Alendronate | | 14 | | 5.0 | | 9.0 | | 65.5 | | 6/8 | | 4 (28.6) | | 0.875 | |
| **Funda Tascioglu 2004** | |  | |  | |  | |  | |  | |  | |  | |
| Alendronate | | 22 | | 8.00±1.77 | | 48.00±21.12 | | 55.67±6.67 | | 0/22 | | 22(100.0) | | 0.69±0.07 | |
| Calcitonin | | 24 | | 7.58±2.04 | | 54.48±27.36 | | 58.13±6.51 | | 0/24 | | 24(100.0) | | 0.68±0.07 | |
| **Shegeki Yamada 2007** | |  | |  | |  | |  | |  | |  | |  | |
| Risedronate | | 6 | | 3.5±1.7 | | 33.3±5.7 | | 69.2±6.0 | | 0/6 | | 6（100） | | 0.64±0.10 | |
| Alfacalcidol | | 6 | | 3.8±2.8 | | 25.6±12.3 | | 72.0±8.7 | | 0/6 | | 6（100） | | 0.64±0.10 | |
| **Jese S. Siffledeen 2005** | |  | |  | |  | |  | |  | |  | |  | |
| Etidronate | | 72 | | NM | | 5.6 ±1.9 | | 40.0±12.1 | | 38/34 | | NM | | 0.94±0.10 | |
| Placebo | | 71 | | NM | | 5.4 ±1.6 | | 40.1±14.1 | | 34/37 | | NM | | 0.91±0.11 | |
| **Kenneth G. Saag 2007** | |  | |  | |  | |  | |  | |  | |  | |
| alendronate | | 214 | | 7.8 | | 14.4 | | 57.3±14.0 | | 41/173 | | 143 (82.7) | | 0.85±0.13 | |
| teriparatide | | 214 | | 7.5 | | 18 | | 56.1±13.4 | | 42/172 | | 134 (77.9) | | 0.85±0.13 | |
| **Benito R. Losada 2008** | |  | |  | |  | |  | |  | |  | |  | |
| alendronate | | 32 | | 7.5±1.7 | | 5.3±2.9 | | 54.9±4.5 | | 5/27 | | NM | | 0.8 ±0.05 | |
| teriparatide | | 29 | | 8.8±1.9 | | 2.7±3.2 | | 52.5 ±5.0 | | 5/24 | | NM | | 0.8 ±0.05 | |
| **Alan L. Burshell 2009** | |  | |  | |  | |  | |  | |  | |  | |
| alendronate | | 77 | | 8.0 | | 16.8 | | 60.6±2.5 | | 17/60 | | 50（64.9） | | −2.7±0.1 | |
| teriparatide | | 80 | | 7.5 | | 14.4 | | 56.1±2.6 | | 13/67 | | 41（51.3） | | −2.5±0.1 | |
| **Jean-Pierre 2009** | |  | |  | |  | |  | |  | |  | |  | |
| alendronate | | 192 | | 10.1±0.7 | | 5.1 ± 0.5 | | 57.1±1.0 | | NM | | NM | | 0.85±0.01 | |
| teriparatide | | 195 | | 9.4±0.4 | | 5.2 ± 0.6 | | 55.8±1.0 | | NM | | NM | | 0.85±0.01 | |
| **B. L. Langdahl 2009** | |  | |  | |  | |  | |  | |  | |  | |
| **alendronate** | |  | |  | |  | |  | |  | |  | |  | |
| Postmenopausal | | 143 | | 7.3 | | 26.4 | | 62.1±1.2 | | 0/143 | | 143(100) | | −2.7±0.1 | |
| Premenopausal | | 30 | | 10.0 | | 10.8 | | 35.8±2.1 | | 0/30 | | 0 | | −2.6±0.2 | |
| Men | | 41 | | 10.0 | | 25.2 | | 59.7±1.9 | | 41/0 | | 0 | | −2.3±0.2 | |
| **teriparatide** | |  | |  | |  | |  | |  | |  | |  | |
| Postmenopausal | | 134 | | 7 | | 31.2 | | 61.9±1.2 | | 0/134 | | 134(100) | | −2.7±0.1 | |
| Premenopausal | | 37 | | 8 | | 21.6 | | 40.0±1.9 | | 0/37 | | 0 | | −2.4±0.2 | |
| Men | | 42 | | 10 | | 27.6 | | 55.5±1.9 | | 42/0 | | 0 | | −2.3±0.2 | |
| **Kenneth G. Saag 2009** | |  | |  | |  | |  | |  | |  | |  | |
| alendronate | | 214 | | ≥5 | | 24 | | 57.3±14.0 | | 41/173 | | 143 (66.8) | | 0.864±0.014 | |
| teriparatide | | 214 | | ≥5 | | 27.6 | | 56.1±13.4 | | 42/172 | | 134 (62.6) | | 0.863±0.014 | |
| **Kenneth G Saag 2016** | |  | |  | |  | |  | |  | |  | |  | |
| alendronate | | 214 | | 7.5 | | 48 | | 57±14 | | 41/173 | | NM | | -2.5 ± 0.1 | |
| teriparatide | | 214 | | 7.5 | | 27.6 | | 56±13 | | 42/172 | | NM | | -2.4 ± 0.1 | |
| **Kenneth G. Sagg 1998** | |  | |  | |  | |  | |  | |  | |  | |
| placebo | | 159 | | 10 | | NM | | 54±15 | | 52/107 | | 67 (42.1) | | 0.95±0.16 | |
| alendronate | | 157 | | 10 | | NM | | 55±15 | | 44/113 | | 83 (52.9) | | 0.93±0.16 | |
| **S.Aubrey.Stoch 2009** | |  | |  | |  | |  | |  | |  | |  | |
| Alendronate | | 114 | | 16.5±11.6 | | 54.6±72.0 | | 51.9±14.4 | | 44/70 | | 29(25.4) | | -0.33±1.37 | |
| Placebo | | 59 | | 15.6±12.0 | | 44.8±63.0 | | 54.6±14.8 | | 28/31 | | 17(28.8) | | 0.38±1.11 | |
| **Jonathan D. Adachi 2000** | |  | |  | |  | |  | |  | |  | |  | |
| Placebo | | 61 | | 20.4 ± 20.7 | | ≥3 | | 54 ± 15 | | 19/42 | | 25(41.0) | | 0.93 ± 0.15 | |
| Alendronate | | 55 | | 17.4 ± 18.0 | | ≥3 | | 53 ± 15 | | 15/40 | | 26(47.3) | | 0.93 ± 0.15 | |
| **Chi Chiu Mok 2010** | |  | |  | |  | |  | |  | |  | |  | |
| Raloxifene | | 57 | | 7.2±6.2 | | 58.1 | | 55.4±7.8 | | 0/57 | | 57(100) | | 0.864±0.136 | |
| Placebo | | 57 | | 6.5±5.5 | | 67.8 | | 55.2±7.6 | | 0/57 | | 57(100) | | 0.848±0.147 | |
| **David M Reid 2009** | |  | |  | |  | |  | |  | |  | |  | |
| Zoledronic acid | | 272 | | 10 | | ≥12 | | 53.2 ±14.0 | | 87/185 | | 118（43.4） | | –1.34±1.34 | |
| Risedronate | | 273 | | 10 | | ≥12 | | 52.7 ±13.7 | | 90/183 | | 117（42.9） | | –1.40±1.28 | |
| **Philip N Sambrook 2011** | |  | |  | |  | |  | |  | |  | |  | |
| Zoledronic acid | | 75 | | 15.3±13.11 | | ＞3 | | 57.2±14.73 | | 75/0 | | 0 | | 0.929±0.152 | |
| Risedronate | | 77 | | 15.5±12.12 | | ＞3 | | 55.7±13.95 | | 77/0 | | 0 | | 0.920±0.139 | |
| **Claus-C.Glüer 2012** | |  | |  | |  | |  | |  | |  | |  | |
| Teriparatide | | 45 | | 8.8 | | 85.2 | | 57.5±12.8 | | NM | | NM | | -2.48 | |
| Risedronate | | 47 | | 8.8 | | 58.8 | | 55.1±15.5 | | NM | | NM | | -2.33 | |
| **Kenneth G. Saag 2019** | |  | |  | |  | |  | |  | |  | |  | |
| Risedronate | | 252 | | 11.1 ± 7.69 | | ≥3 | | 61.3±11.1 | | 67/185 | | 157（62.3） | | –1.96 ± 1.38 | |
| Denosumab | | 253 | | 12.3 ± 8.09 | | ≥3 | | 61.5±11.6 | | 68/185 | | 159（62.8） | | –1.92 ± 1.38 | |
| **R. Eastell 1999^d^** | |  | |  | |  | |  | |  | |  | |  | |
| Placebo | | 40 | | 812±286 | | 199.2 | | 65.0±6.3 | | 0/40 | | 40（100） | | 0.76±0.13 | |
| Risedronate | | 40 | | 810±298 | | 162 | | 64.5±7.2 | | 0/40 | | 40（100） | | 0.80 ± 0.13 | |
| **David M. Reid 1999** | |  | |  | |  | |  | |  | |  | |  | |
| Placebo | | 96 | | 15±13 | | 62±72 | | 59±12 | | 36/60 | | 53±55.2 | | -1.7±1.5 | |
| Risedronate | | 100 | | 15±12 | | 57±58 | | 58±12 | | 36/64 | | 55±55.0 | | -1.7±1.6 | |
| **Sonsoles Guadalix 2011^d^** | |  | |  | |  | |  | |  | |  | |  | |
| Risedronate | | 45 | | 3931.2±2129.4 | | 12 | | 57.9 ± 6.5 | | 32/13 | | 13（28.9） | | 0.792 ± 0.104 | |
| Placebo | | 44 | | 4584.0±2638.6 | | 12 | | 54.6 ± 8.8 | | 38/6 | | 4（9.1） | | 0.844 ± 0.089 | |
| **Naohiko Fujii 2006** | |  | |  | |  | |  | |  | |  | |  | |
| Placebo | | 37 | | 10.6±5.1 | | 6.5±8.1 | | 42.2±16.5 | | 16/21 | | 6(16.2) | | 1.094±0.119 | |
| risedronate | | 40 | | 9.9±5.0 | | 5.2±6.3 | | 40.0±16.3 | | 15/25 | | 6(15.0) | | 1.054±0.137 | |
| **A Rmando T Orres 2004** | |  | |  | |  | |  | |  | |  | |  | |
| Calcitriol | | 45 | | 10 | | 12 | | 46.7±12.2 | | 37/8 | | 3（6.7） | | 1.02 ± 0.12 | |
| Placebo | | 41 | | 10 | | 12 | | 51.1±11.9 | | 30/11 | | 7（17.1） | | 0.98 ± 0.12 | |
| **Toshio Matsumoto 2020** | |  | |  | |  | |  | |  | |  | |  | |
| Eldecalcitol | | 178 | | 10.3±9.0 | | ＞3 | | 58.5±16.2 | | 62/116 | | 72 (62.1) | | − 0.70±1.39) | |
| Alfacalcidol | | 182 | | 9.5±7.7 | | ＞3 | | 58.4±15.7 | | 59/123 | | 75 (61.0) | | − 0.54±1.39) | |
| **J. D. Ringe 1999** | |  | |  | |  | |  | |  | |  | |  | |
| Alfacalcidol | | 43 | | 9.7 | | 70.8 | | 60.6 | | 15/28 | | NM | | −3.28 | |
| vitamin D | | 42 | | 9.6 | | 49.2 | | 60.7 | | 15/27 | | NM | | −3.25 | |
| **J. D. Ringe 2003** | |  | |  | |  | |  | |  | |  | |  | |
| Alfacalcidol | | 103 | | 8.0 | | 36 | | 60.1±9.8 | | 38/65 | | NM | | 3.26±0.57 | |
| vitamin D | | 101 | | 7.5 | | 36 | | 60.3±9.9 | | 36/65 | | NM | | 3.25±0.39 | |
| **Satoshi Soen 2019** | |  | |  | |  | |  | |  | |  | |  | |
| Minodronate | | 40 | | 7.53 ± 6.57 | | 44.0 ± 48.3 | | 62.0±13.5 | | 17/23 | | NM | |  | |
| Placebo | | 42 | | 7.62 ± 5.74 | | 41.5 ± 42.5 | | 61.3 ± 9.6 | | 23/19 | | NM | | 93.1 ± 16.0 | |
| **P.Pitt 1997** | |  | |  | |  | |  | |  | |  | |  | |
| etidronate | | 26 | | 8.2±4.2 | | 104 | | 58.9±13.7 | | 10/16 | | NM | | 0.74±0.12 | |
| placebo | | 23 | | 7.2±4.0 | | 104 | | 59.2±10.8 | | 9/14 | | NM | | 0.76±0.11 | |
| **Christian Roux 1998** | |  | |  | |  | |  | |  | |  | |  | |
| placebo | | 58 | | ≥7.5 | | ≥12 | | 59.0±13.6 | | 20/38 | | 30（51.7） | | 0.924±0.156 | |
| etidronate | | 59 | | ≥7.5 | | ≥12 | | 58.5±13.9 | | 22/37 | | 27（45.8） | | 0.897±0.158 | |
| **Jacques P. Brown 2001** | |  | | 22.7 ± 21.7 | |  | |  | |  | |  | |  | |
| placebo | | 61 | | 22.7 ± 21.7 | | ≥52 | | 60 ± 17 | | 24/37 | | 29（47.5） | | NM | |
| etidronate | | 53 | | 20.5 ± 22.2 | | ≥52 | | 64 ± 13 | | 17/36 | | 29（54.7） | | NM | |
| **I.Garcia-Delgado 1996** | | SE | |  | |  | |  | |  | |  | |  | |
| Calcitonin | | 13 | | NM | | NM | | 55.9±1.63 | | 13/0 | | NM | | 0.854 ± 0.069 | |
| Etidronate | | 14 | | NM | | NM | | 52.7±1.82 | | 14/0 | | NM | | 0.871 ± 0.091 | |
| **Y. Boutsen 1997** | |  | |  | |  | |  | |  | |  | |  | |
| Pamidronate | | 14 | | 31.2±23.8 | | NM | | 60±16 | | 3/11 | | 3(21.4) | | 0.857±0.118 | |
| Calcium | | 13 | | 28.1±23.8 | | NM | | 61±12 | | 2/11 | | 2(15.4) | | 0.960±0.161 | |
| **Y. Boutsen 2000** | |  | |  | |  | |  | |  | |  | |  | |
| Pamidronate | | 9 | | ≥10 | | ≥3 | | 59±21 | | 4/5 | | 4（44.4） | | 0.965±0.161 | |
| Calcium | | 9 | | ≥10 | | ≥3 | | 57±18 | | 4/5 | | 4（44.4） | | 0.963±0.173 | |
| **T. Bianda 2000^d^** | |  | |  | |  | |  | |  | |  | |  | |
| Calcitonin | | 12 | | 14800 ± 1200 | | 12 | | 54.5 ± 1.0 | | 11/1 | | NM | | 0.97 ± 0.04 | |
| Pamidronate | | 14 | | 13800 ± 1700 | | 12 | | 51.1 ± 3.0 | | 13/1 | | NM | | 1.01 ± 0.03 | |
| **Se Hwa Kim 2003** | |  | |  | |  | |  | |  | |  | |  | |
| Placebo | | 20 | | NM | | NM | | 48±18 | | 9/11 | | 7(35.0) | | 0.897±0.193 | |
| Pamidronate | | 25 | | NM | | NM | | 49±15 | | 14/11 | | 7(28.0) | | 0.864±0.185 | |
| **A Nzeusseu Toukap 2005** | |  | |  | |  | |  | |  | |  | |  | |
| Pamidronate | | 16 | | ≥7.5 | | ≥12 | | 30.5±7.4 | | NM | | NM | | 0.954±0.108 | |
| Placebo | | 14 | | ≥7.5 | | ≥12 | | 25.3±9.2 | | NM | | NM | | 0.974±0.147 | |
| **B. Frediani 2003** | |  | |  | |  | |  | |  | |  | |  | |
| Clodronate | | 84 | | 8.4 ± 3.2 | | NM | | 61.1±12.2 | | 0/84 | | 63(75.0) | | 0.99 ± 0.18 | |
| Placebo | | 79 | | 8.9 ± 4.1 | | NM | | 62.4±13.4 | | 0/79 | | 61(77.2) | | 0.98 ± 0.16 | |
| **Vered Abitbol 2007** | |  | |  | |  | |  | |  | |  | |  | |
| Clodronate | | 33 | | 15.0 | | 12 | | 30 | | 16/17 | | NM | | -1.3±1.10 | |
| Placebo | | 34 | | 14.0 | | 12 | | 30 | | 14/20 | | NM | | -1.2±1.33 | |
| **CC Mok 2013** | |  | |  | |  | |  | |  | |  | |  | |
| Raloxifene | | 30 | | 7.9±7.4 | | 89.2±71 | | 52.5±6.7 | | 0/30 | | 30(100) | | 0.883±0.125 | |
| Placebo | | 32 | | 5.8±2.6 | | 84.7±65 | | 52.5±6.8 | | 0/32 | | 32(100) | | 0.886±0.134 | |
| **M Hakala 2012** | |  | |  | |  | |  | |  | |  | |  | |
| Ibandronate | | 68 | | 6.71±2.71 | | 40±54 | | 64±8 | | 0/68 | | 68(100) | | 1.128±0.11 | |
| Placebo | | 72 | | 6.67±2.79 | | 44±66 | | 63±7 | | 0/72 | | 72(100) | | 1.146±0.15 | |
| **W.F.Lems 1997** | |  | |  | |  | |  | |  | |  | |  | |
| Placebo | | 24 | | 21.2±17.3 | | ≥6 | | 53±15 | | 10/14 | | 8(33.3) | | 1.043±0.183 | |
| NaF | | 20 | | 14.6±10.5 | | ≥6 | | 49±17 | | 7/13 | | 4(20.0) | | 1.014±0.131 | |
| **Willem F Lems 1997** | |  | |  | |  | |  | |  | |  | |  | |
| Placebo | | 24 | | 16.9±19.8 | | ≥6 | | 60±17 | | 9/15 | | 10（41.7） | | 0.944±0.167 | |
| NaF | | 23 | | 10.6±4.2 | | ≥6 | | 56±17 | | 5/18 | | 15（65.2） | | 0.804±0.142 | |
| **G.Guaydier-Souquibres 1995** | |  | |  | |  | |  | |  | |  | |  | |
| Monofluorophosphate | | 15 | | 15.9±9.4 | | 56.4±39.6 | | 15.9±9.4 | | 12/3 | | NM | | 0.910±0.155 | |
| Placebo | | 13 | | 20.4±16.2 | | 88.8±90.0 | | 20.4±16.22 | | 9/4 | | NM | | 0.925 ±0.129 | |
| **R. Rizzoli 1994^c^** | |  | |  | |  | |  | |  | |  | |  | |
| Monofluorophosphate | | 25 | | 18.2±2.3 | | 111.6±20.4 | | 50.6±3.2 | | 13/12 | | 9(36.0) | | - 1.52±0.19 | |
| Placebo | | 23 | | 12.1±1.1 | | 90.0±21.6 | | 51.6±3.0 | | 10/13 | | 9(39.1) | | - 1.19±0.18 | |

*All Patients received supplements of calcium (1000 mg/d) and vitamin D (800 IU/d)

^a^If there is no special instructions, all values are mean ± SD

^b^ prednisone or equivalent

^c^values are mean±SE

^d^12 months cumulative dose of prednison(mean±SD)

BMD = bone mineral density

LS = lumbar spine

GC = glucocorticoid

M = male

F = female

NM = not mentioned
