# Supplementary material for: Efficacy and safety of 18 anti-osteoporotic drugs in the treatment of patients with osteoporosis caused by glucocorticoid: A network meta-analysis of randomized controlled trials
Source: PLoS One. 2020 Dec 16;15(12):e0243851. doi: 10.1371/journal.pone.0243851 (PMC7743932; doi:10.1371/journal.pone.0243851)
Supplement: S2 Table — (DOCX) [file pone.0243851.s002.docx]

**Table 2 SUCRA ranking**

| Rank or Outcomes | 1 | 2 | 3 | 4 | 5 | 6 | 7 | 8 | 9 | 10 | 11 | 12 | 13 | 14 | 15 | 16 | 17 | 18 | 19 |
| --- | --- | --- | --- | --- | --- | --- | --- | --- | --- | --- | --- | --- | --- | --- | --- | --- | --- | --- | --- |
| LS BMD | RAL | PAM | DEN | CLO | MFP | NaF | TPTD | Ca | CT | MIN | ALE | ELD | IBA | ALF | ZOL | ETI | RIS | PLA | VD3 |
| TH BMD | DEN | PAM | RAL | Ca | TPTD | ALE | IBA | RIS | CT | ZOL | ETI | ELD | MIN | PLA | ALE | NaF |  |  |  |
| VF | TPTD | PAM | RAL | ETI | VD3 | CT | ALE | CLO | DEN | RIS | ELD | NaF | ZOL | PLA | ALF | MFP | MIN |  |  |
| non-VF | IBA | ALE | ETI | ALF | TPTD | ELD | PLA | VD3 | RIS | DEN |  |  |  |  |  |  |  |  |  |
| AE | CT | ALF | VD3 | MIN | ELD | ALE | TPTD | ETI | CLO | ZOL | RAL | Ca | DEN | MFP | PLA | RIS | PAM | IBA |  |

SUCRA= the surface under the cumulative ranking curve; LS= lumbar spine; TH= total hip; BMD= bone mineral density; RAL= raloxifene; PAM= pamidronate DEN= denosumab;

CLO= clodronate; MFP= monofluorophosphate; NaF= sodium fluoride; TPTD= teriparatide; Ca= calcium; CT= calcitonin; MIN= minodronate; ALE=alendronate; ELD= eldecalcitol

IBA= ibandronate; ALF= alfacalcidol; ZOL= zoledronic acid; ETI= etidronate; RIS= risedronate; PLA= placebo; VD3= Vitamin D_3_; VF= vertebral fractures; non-VF= non-vertebral fractures

AE= adverse events
