# Supplementary figures and images for: Efficacy and safety of 18 anti-osteoporotic drugs in the treatment of patients with osteoporosis caused by glucocorticoid: A network meta-analysis of randomized controlled trials
Source: PLoS One. 2020 Dec 16;15(12):e0243851. doi: 10.1371/journal.pone.0243851 (PMC7743932; doi:10.1371/journal.pone.0243851)

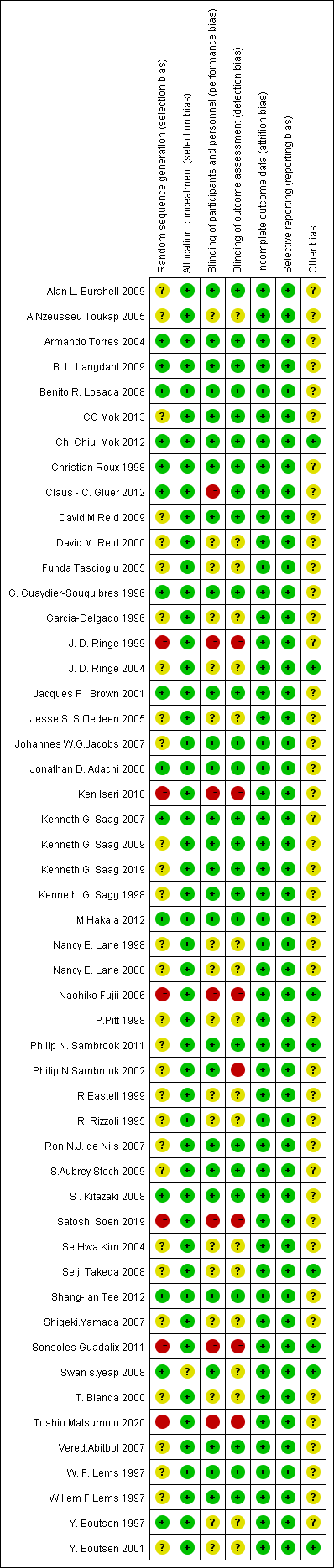

Supplement: S2 File — (PNG) [file pone.0243851.s004.png]

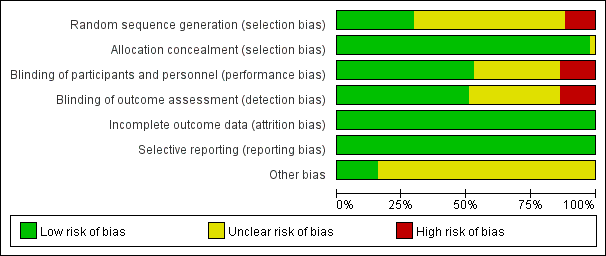

Supplement: S3 File — (PNG) [file pone.0243851.s005.png]
