# Supplementary material for: Efficacy and safety of 18 anti-osteoporotic drugs in the treatment of patients with osteoporosis caused by glucocorticoid: A network meta-analysis of randomized controlled trials
Source: PLoS One. 2020 Dec 16;15(12):e0243851. doi: 10.1371/journal.pone.0243851 (PMC7743932; doi:10.1371/journal.pone.0243851)
Supplement: S4 File — (DOCX) [file pone.0243851.s006.docx]

The search strategy for PubMed is as follows:

Search ((((((((((((glucocorticoid[Title/Abstract]) OR glucocorticoids[Title/Abstract]) OR corticoid[Title/Abstract]) OR corticoids[Title/Abstract]) OR corticosteroids[Title/Abstract]) OR corticosteroid[Title/Abstract]) OR methylprednisolone[Title/Abstract]) OR prednisone [Title/Abstract]) OR prednisolone [Title/Abstract]) OR hydrocortisone[Title/Abstract]) OR triamcinolone[Title/Abstract]) OR dexamethasone [Title/Abstract]) AND osteoporosis[Title/Abstract] Filters: Randomized Controlled Trial; Full text; Humans Sort by: [pubsolr12]

The search strategy for Embase is as follows:

(glucocorticoid*:ti OR corticosteroid*:ti OR methylprednisolone:ti OR prednisone:ti OR prednisolone:ti OR hydrocortisone:ti OR triamcinolone:ti OR dexamethasone:ti OR corticoid*:ti) AND osteoporosis:ti AND 'randomized controlled trial*':ti,ab,kw

The search strategy for Cochrane library is as follows:

Trials matching glucocorticoid* or corticosteroid* or methylprednisolone or prednisone or prednisolone or hydrocortisone or triamcinolone or dexamethasone or corticoid* in Record Title AND randomized controlled trial* in Title Abstract Keyword AND osteoporosis in Record Title - in Trials (Word variations have been searched)
