# Supplementary material for: Efficacy and safety of 18 anti-osteoporotic drugs in the treatment of patients with osteoporosis caused by glucocorticoid: A network meta-analysis of randomized controlled trials
Source: PLoS One. 2020 Dec 16;15(12):e0243851. doi: 10.1371/journal.pone.0243851 (PMC7743932; doi:10.1371/journal.pone.0243851)
Supplement: S5 File — (DOCX) [file pone.0243851.s007.docx]

Risk of bias summary

| Studies | Selection bias (random sequence generation and allocation concealment) | Performance bias (blinding of participants and personnel) | Detection bias (blinding of outcome assessment) | Attrition bias (incomplete outcome data) | Reporting bias (selective reporting) | Other potential bias (other biases not included in the above five) |
| --- | --- | --- | --- | --- | --- | --- |
| Swan s. yeap 2008 | Unclear: randomisation and allocation concealment not specified clearly | Low | Low | Low | Low | Low |
| Benito R. Losada 2009 | Low: random-number table  Unclear: allocation concealment | Low | Low | Low | Low | Low |
| Kenneth G. Saag 2007 | Low: computer-generated ran  domization procedure  allocation concealment | Low | Low | Low | Low | Low |
| Ron 2006 | Low: computer-generated ran  domization procedure  Low:  allocation concealment | Low | Low | High: high proportion of missing data (38/210) | Low | Low |
| S.Kitazaki 2008 | Low: envelope method;  Low: allocation concealment | Unclear: blinding not reported | Unclear: blinding not reported | Low | Low | Low |
| Alan L. Burshell 2010 | Unclear: randomisation and allocation concealment not reported | Low | Low | Low | Low | Low |
| Seiji Takeda 2008 | Unclear: randomisation not reported;  Unclear: allocation concealment not reported | Unclear: blinding not reported | Unclear: blinding not reported | Low | Low | Low |
| Kenneth G. Saag 2009 | Unclear: randomisation not reported;  Low: allocation concealment | Low | Low | Low | Low | Low |
| S.Aubrey Stoch 2009 | Unclear: randomisation not reported;  Low: allocation concealment | Low | Low | Low | Low | Low |
| Philip N Sambrook 2002 | Unclear: randomisation and allocation concealment not reported | High: This was an open-label study | High: This was an open-label study | Low | Low | Low |
| Johannes W.G.Jacobs 2007 | Unclear: randomisation not reported;  Low: allocation concealment | Low | Low | Low | Low | Low |
| Kenneth G. Saag 2007 | Unclear: randomisation and allocation concealment not reported | Low | Low | Low | Low | Low |
| B. L. Langdahl 2009 | Low: computer-generated randomisation and adequate concealment | Low | Low | Low | Low | Low |
| Ken Iseri 2018 | Low: random-number table and adequate concealment | High: This was an open-label study | High: This was an open-label study | Low | Low | Low |
| Funda Tascioglu 2005 | Low: computer-generated randomisation and adequate concealment | High: This was an open-label study | High: This was an open-label study | Low | Low | Low |
| Jonathan D. Adachi 2000 | Low: computer-generated randomisation and adequate concealment | Low | Low | Low | Low | Low |
| Shang-Ian Tee 2012 | Unclear: randomisation and allocation concealment not reported | Low | Low | Low | Low | Low |
| Y. Boutsen 1997 | Low: computer-generated ran  domization procedure  Unclear: concealment not reported | Unclear: blinding not reported | Unclear: blinding not reported | Low | Low | Low |
| Philip N. Sambrook 2011 | Unclear: randomisation not reported;  Low: allocation concealment | Low | Low | Low | Low | Low |
| Shigeki.Yamada 2007 | Unclear: randomisation and allocation concealment not reported | Unclear: blinding not reported | Unclear: blinding not reported | Low | Low | Low |
| Claus - C. Glüer 2012 | Low: computer-generated randomisation and adequate concealment | High: This was an open-label study | High: This was an open-label study | Low | Low | Low |
| Kenneth G. Saag 2019 | Low: computer-generated randomisation and adequate concealment | Low | Low | Low | Low | Low |
| Sonsoles Guadalix 2011 | Low: computer-generated randomisation and adequate concealment | High: This was an open-label study | High: This was an open-label study | Low | Low | Low |
| David M. Reid 2000 | Unclear: randomisation and allocation concealment not reported | Unclear: blinding not reported | Unclear: blinding not reported | Low | Low | Low |
| R.Eastell 1999 | Unclear: randomisation not reported;  Low: allocation concealment | Low | Low | Low | Low | Low |
| Naohiko Fujii 2006 | Low: computer-generated randomisation and adequate concealment | Low | Low | Low | Low | Low |
| P.Pitt 1998 | Unclear: randomisation not reported;  Low: allocation concealment | Low | Low | Low | Low | Low |
| Vered.Abitbol 2007 | Unclear: randomisation not reported;  Low: adequate concealment | Low | Low | Low | Low | Low |
| Garcia-Delgado 1996 | Unclear: randomisation not reported;  Low: adequate concealment | Unclear: blinding not reported | Unclear: blinding not reported | Low | Low | Low |
| CC Mok 2013 | Unclear: randomisation not reported;  Low: adequate concealment | Low | Low | Low | Low | Low |
| W. F. Lems 1997 | Unclear: randomisation not reported;  Low: adequate concealment | Low | Low | Low | Low | Low |
| Se Hwa Kim 2004 | Unclear: randomisation and allocation concealment not reported | Unclear: blinding not reported | Unclear: blinding not reported | Low | Low | Low |
| Toshio Matsumoto 2020 | Unclear: randomisation and allocation concealment not reported | High: This was an open-label study | High: This was an open-label study | Low | Low | Low |
| G. Guaydier-Souquibres 1996 | Unclear: randomisation and allocation concealment not reported | Low | Low | Low | Low | Low |
| Willem F Lems 1997 | Unclear: randomisation not reported;  Low: adequate concealment | Low | Low | Low | Low | Low |
| Satoshi Soen 2019 | Unclear: randomisation not reported;  Low: adequate concealment | High: This was an open-label study | High: This was an open-label study | Low | Low | Low |
| M Hakala 2012 | Unclear: randomisation not reported;  Low: adequate concealment | Low | Low | Low | Low | Low |
| A Nzeusseu Toukap 2005 | Unclear: randomisation and allocation concealment not reported | Unclear: blinding not reported | Unclear: blinding not reported | Low | Low | Low |
| Jacques P . Brown 2001 | Unclear: randomisation not reported;  Low: adequate concealment | Low | Low | Low | Low | Low |
| T. Bianda 2000 | Unclear: randomisation not reported;  Low: adequate concealment | Unclear: blinding not reported | Unclear: blinding not reported | Low | Low | Low |
| Y. Boutsen 2001 | Unclear: randomisation not reported;  Low: adequate concealment | Low | Low | Low | Low | Low |
| Nancy E. Lane 2000 | Unclear: randomisation not reported;  Low: adequate concealment | Unclear: blinding not reported | Unclear: blinding not reported | Low | Low | Low |
| Nancy E. Lane 1998 | Unclear: randomisation not reported;  Low: adequate concealment | Unclear: blinding not reported | Unclear: blinding not reported | Low | Low | Low |
| CC Mok 2013 | Low: randomisation code  Low: adequate concealment | Low | Low | Low | Low | Low |
| Christian Roux 1998 | Unclear: randomisation not reported;  Low: adequate concealment | Low | Low | Low | Low | Low |
| Jesse S. Siffledeen 2005 | Unclear: randomisation not reported;  Low: adequate concealment | Unclear: blinding not reported | Unclear: blinding not reported | Low | Low | Low |
| R. Rizzoli 1995 | Unclear: randomisation not reported;  High: no concealment | Unclear: blinding not reported | Unclear: blinding not reported | Low | Low | Low |
| J. D. Ringe 2004 | Unclear: randomisation and concealment not reported | Unclear: blinding not reported | Unclear: blinding not reported | Low | Low | Low |
| J. D. Ringe 1999 | Unclear: randomisation and concealment not reported | Low | Low | Low | Low | Low |
| Armando Torres 2004 | Unclear: randomisation not reported;  Low: adequate concealment | Low | Low | Low | Low | Low |
| Shang-Ian Tee 2012 | Unclear: randomisation not reported;  Low: adequate concealment | Low | Low | Low | Low | Low |
